# Engineered Anti‐Senescence Trachea With Post‐Transplanted Regenerative Homeostasis

**DOI:** 10.1002/advs.202507186

**Published:** 2025-07-06

**Authors:** Ziyin Pan, Hai Tang, Lanlan Wang, Qingfeng Bai, Yi Chen, Runfeng Cao, Weikang Lin, Lei Wang, Yulong Hu, Guofang Zhao, Minglei Yang, Weiyan Sun, Kun Zhang, Dawei Li, Chang Chen

**Affiliations:** ^1^ Department of Thoracic Surgery, Shanghai Pulmonary Hospital Tongji University School of Medicine Shanghai 200433 China; ^2^ Shanghai Engineering Research Center of Lung Transplantation, Shanghai Pulmonary Hospital Tongji University Shanghai 200433 China; ^3^ Nonwoven Technology Laboratory College of Textile Science and Engineering Jiangnan University Wuxi 214122 China; ^4^ Central Laboratory and Department of Medical Ultrasound, Sichuan Academy of Medical Sciences, Sichuan Provincial People's Hospital, School of Medicine University of Electronic Science and Technology of China Sichuan 610072 China; ^5^ College of Fashion Henan Institute of Science and Technology Xinxiang 453003 China; ^6^ Department of Thoracic Oncology Surgery, Fujian Cancer Hospital Clinical Oncology School of Fujian Medical University Fuzhou 350014 China; ^7^ Department of Cardiothoracic Surgery Ningbo No.2 Hospital Ningbo 315010 China; ^8^ Ningbo Institute of Life and Health Industry University of Chinese Academy of Sciences Ningbo 315016 China

**Keywords:** long‐segment tracheal defect, post‐transplanted dysfunction, senescence, tissue‐engineered trachea

## Abstract

Post‐transplanted dysfunction remains the key unsolved challenge to construct bioengineered complex organs. Herein, by analyzing the bioengineered trachea, it is found that multiple tissue senescences occur, and poor endogenous cellular contact and exogenous immune microenvironment dysregulation are identified as two crucial factors during senescence. Therefore, a Hebe engineered trachea (Hebe‐ET) with a ring–ring structure and dual anti‐senescence designs is proposed for maintaining regenerative homeostasis after transplantation. First, in the cartilage rings, a fiber‐film structural scaffold is designed to promote close‐packed cellular contact, significantly reducing senescent P21+ chondrocytes. Furthermore, between the cartilage rings are distributed fibrous connective tissue rings, in which the loaded quercetin induces an immune cascade to restrain senescence in multiple cells, including chondrocytes, endothelial cells, and fibroblasts via mitochondrion‐targeted oxidative stress scavenging, promoting the development of the full tracheal components in vivo. Based on these designs, 12 weeks after orthotopic transplantation, the Hebe‐ET achieves sustainable youth and develops a natural‐like structure with mature cartilage phenotype, reconstructed vascular network, and epithelium coverage. The mechanical property exceeds that of the natural trachea, and 87.5% of animals survived. This study first reveals the necessity of anti‐senescence design in the fabrication of complex organ substitutes and proposes an effective engineering strategy for segmental trachea reconstruction.

## Graphical abstract

Bioengineered trachea undergoes post‐transplanted senescence, which is found in this study for the first time. Hebe‐ET is designed with dual anti‐senescence strategies: fiber‐film scaffold in cartilage rings for close‐packed cell interaction, and quercetin‐loaded fibrous rings for immune modulation. After transplantation, Hebe‐ET develops a natural‐like structure, with enhanced mechanical properties and normal tracheal functions.

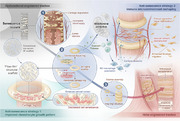

## 1 Introduction

Senescence disrupts normal cellular functions and, as a cellular fate, is characterized by permanent cell cycle arrest and the secretion of large amounts of harmful factors. In addition to the cellular level, with the accumulation of senescence, cell proliferation decreases, and tissue regenerative homeostasis is disturbed; thus, tissue repair is impaired.^[^ ^1^, ^2^ ^]^ The occurrence and progression of a series of clinically important degenerative diseases, such as osteoarthritis^[^ ^3^ ^]^ and chronic tendon injuries^[^ ^4^ ^]^ are related to senescence, which has also been proven to affect the physiological integrity and regenerative potential of grafts e.g., allogeneic kidneys^[^ ^5^ ^]^ and artificial vascular grafts^[^ ^6^ ^]^ in vivo and ultimately determines graft survival conditions. In the field of tracheal transplantation, although engineered tracheae mimicking natural structures are being developed, after orthotopic transplantation, the tracheal function was found to be decreased to varying degrees with fibroplasia, cartilage degradation, and loss of epithelial regeneration^[^ ^7^, ^8^ ^]^; however, whether these degenerative changes are related to senescence remains unknown.

Therefore, to detect the relationship between post‐transplantation functional loss and tissue senescence in tracheal substitutes and thus propose a solution, a dysfunctional engineered trachea constructed from a conventional silk fibroin (SF) scaffold was analyzed (a trachea with a “ring–ring” structure, and ring‐shaped SF scaffolds alternately stacked with chondrocyte rings formed by inoculating chondrocytes on the scaffold). SF was chosen because of its clinical translation potential as a natural source protein with good biocompatibility, biodegradability, and low immunogenicity.^[^ ^9^ ^]^ Derivatives of SF, such as bioabsorbable surgical sutures and ligament grafts, have been approved by the U.S. Food and Drug Administration (FDA) for clinical use.^[^ ^10^ ^]^ After analysis, 1) within the cartilage rings, the accumulation of endogenous senescence in the chondrocytes due to poor cellular contact, and 2) in the fibrous connective tissues between the cartilage rings, the dysregulation of the immune‐senescence cascade was identified as the two key factors. Therefore, for the first time, this study proposed a Hebe (presenting eternal youth) engineered trachea (Hebe‐ET) with targeted dual anti‐senescence designs to achieve long‐term regenerative homeostasis in vivo.

First, in order to optimize the development pattern of chondrocytes, on the original basis, enhanced SF films were introduced, and a hybrid scaffold with a “fiber‐film” structure was constructed. Intercellular communication was proven to be regulated by the growth environment provided by the scaffold, which ultimately affects cell behavior and the fate of regenerated tissue.^[^ ^11^, ^12^, ^13^ ^]^ To promote cell–cell contacts and delay cellular senescence, researchers have attempted to modify the scaffold morphology, such as by adding micro/nano channels or creating grooved surfaces to promote cell attachment and proliferation.^[^ ^14^, ^15^ ^]^ In this study, in the conventional SF scaffold with non‐woven short fibers, chondrocytes had low retention after inoculation and could not form a growth pattern in contact with each other. Cells simply grew along the fibers, which caused morphological stretching and cellular senescence, leading to phenotype loss, and the tracheal cartilage thus degraded and dispersed in vivo. Therefore, enhanced SF films served as a hydrophilic matrix to provide sites for chondrocyte adherence and proliferation, and facilitated cell extension and cell–cell contacts, thus promoting cell growth and development. Meanwhile, the scaffold was endowed with pore interconnectivity in the presence of short fibers, so there was no partition in the whole 3D space, and the chondrocytes could interact with each other, thus slowing the cellular senescence process. In this way, the tracheal cartilage was well developed and maintained stable mechanical support after 12 weeks in vivo.

Furthermore, quercetin was introduced into the fibrous connective tissue rings between the cartilage rings. Quercetin is a polyphenolic flavonoid monomer compound found in natural plants.^[^ ^16^ ^]^ Quercetin, as the main component of “Lung Energy (Mkule)”, has been approved by both the FDA and the current Good Manufacture Practices (cGMPs), for its anti‐inflammatory and free radical scavenging abilities.^[^ ^17^, ^18^, ^19^ ^]^ The inflammatory environment has been shown to increase the cellular senescence phenotype during tissue repair.^[^ ^20^, ^21^ ^]^ During engineered trachea transplantation, factors such as surgical trauma, foreign body reactions, and infections inevitably lead to imbalances in the immune microenvironment^[^ ^22^ ^]^; thus, we can also slow tissue senescence by promoting cell‐microenvironment contacts in engineered trachea construction. Quercetin‐induced macrophage polarization through alleviation of oxidative stress and mitochondrion‐targeted protective effects, resulting in the rejuvenation of chondrocytes, endothelial cells, and fibroblasts via immune niche reshaping. In this way, the regeneration and functional maintenance of the entire tracheal components were significantly enhanced.

In summary, based on the above dual anti‐senescence designs, Hebe‐ET showed no signs of functional loss 12 weeks after transplantation and presented stable mechanical properties, mature cartilaginous phenotype, transmural vascular network in the fibrous connective tissue, and epithelial coverage of the inner wall. This study revealed, for the first time, the critical role of senescence in the long‐term bioactivity maintenance of tracheal substitutes in vivo and provided a reference for the development of complex organ substitutes that meet clinical needs.

## 2 Results

### 2.1 Multiple Tissue Senescence Was Found in the Post‐Transplanted Dysfunctional Trachea (DT)

**Figure 1:**
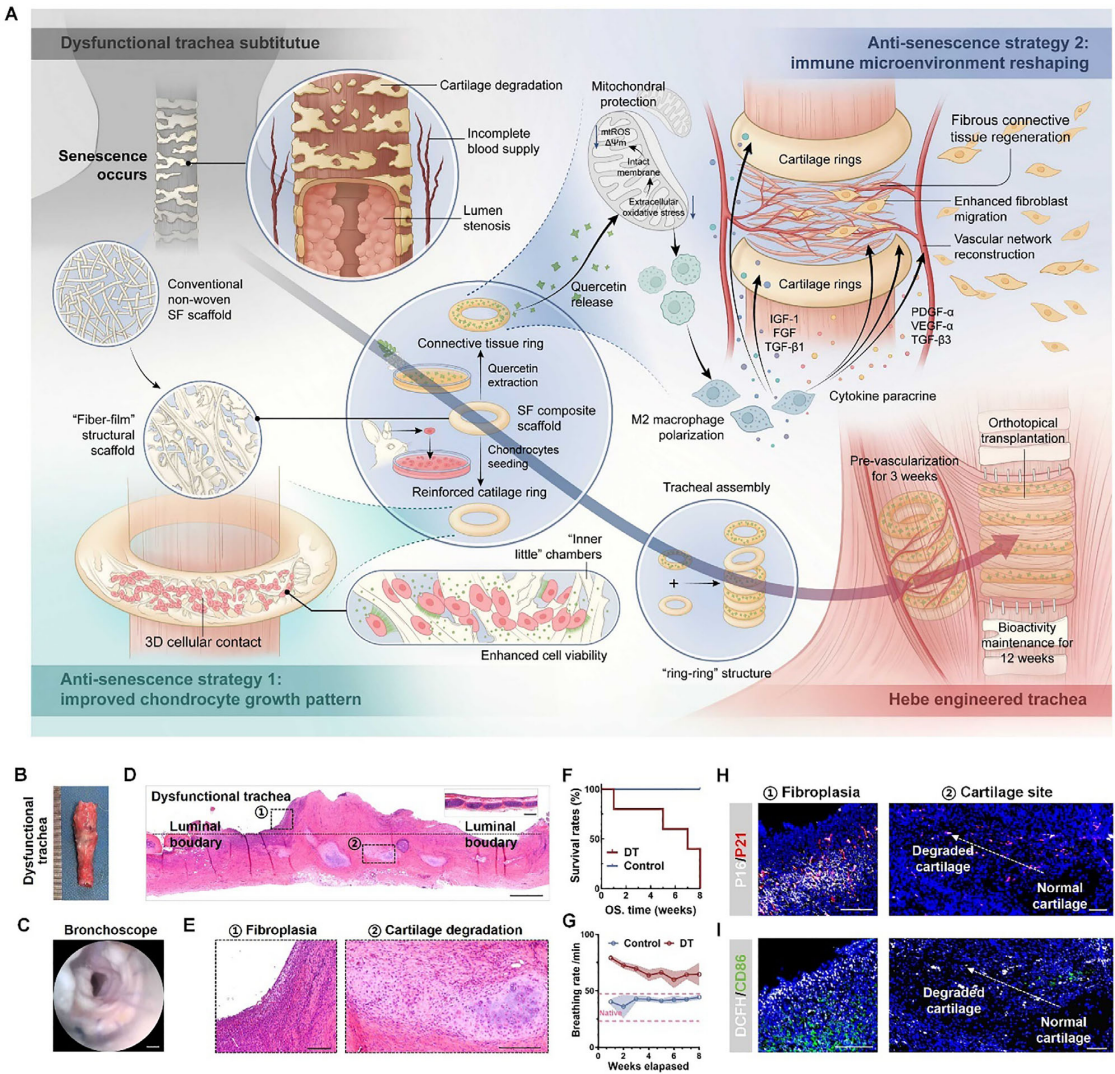
Cause analysis of post‐transplantation tracheal dysfunction. A) Schematic diagram of the preparation of Hebe‐ET and the mechanism of dual anti‐senescence design. B) The gross view of DT after post‐transplantated sampling. C) Bronchoscopy images of DT after transplantation. Scale bar, 1mm. (D,E) H&E tissue section staining of DT. D) The representative longitudinal section of DT and natural trachea (upper right corner). The horizontal dotted line represents the boundary of the normal lumen, and the dotted box represents two representative tissue regions (cartilage degradation and inflammatory granulation proliferation). Scale bar, 1mm. E) Enlarged H&E staining images of the representative area. Scale bar: 200µm. F) Survival curve of experimental rabbits in the DT group, normal rabbits without any treatment as control, *n* = 5 from 5 biologically independent animals. G) The respiratory rate of the DT group rabbits was monitored during the survival period after transplantation, and normal rabbits were used as controls. **(H‐I)** IF staining of senescence markers, as well as inflammatory and oxidative stress markers in two representative tissue regions. The dotted arrow points from normal cartilage to degraded cartilage. Scale bar, 100µm. H) senescence markers (P16, white; P21, red; DAPI, blue). I) Inflammation and oxidative stress markers (CD86, green; DCFH, white; DAPI, blue). DT: dysfunctional trachea; IF: immunofluorescence; DCFH: intra‐tissue reactive oxygen species.

To identify the key driving factors, we analyzed the DT prepared from conventional non‐woven SF fiber scaffolds (C‐SFSs) (**Figure** **1**A). The original ring–ring structure of the trachea disappeared after long‐term transplantation in rabbits, and pus was faintly visible (Figure 1B). The bronchoscopy image showed that the tracheal lumen had obviously narrowed (Figure 1C). H&E staining revealed that the cartilage was severely degraded, only part of the original cartilage was preserved, and inflammatory granulation proliferated toward the inner surface of the lumen, resulting in tracheal stenosis (Figure 1D,E). These results indicated that the structure and function of DTs could not be maintained for a long period after transplantation, and the animals died during the 8‐week observation period after surgery (Figure 1F). Moreover, the respiratory condition of the animals in the surgery group was abnormal, and the respiratory rate was significantly higher compared with the natural state (Figure 1G). Cellular senescence has been shown to affect normal cellular viability and function, leading to various tissue degenerative changes. Therefore, we subsequently performed specific immunofluorescence (IF) staining of tracheal tissues and found that the degraded cartilage, as well as fibrous hyperplasia, presented high expression of P16 and P21, which are senescence indicators (Figure 1H). At the same time, the senescent areas showed immune activation (infiltration of CD86^+^ pro‐inflammatory macrophages) and oxidative stress damage (infiltration of DCFH^+^ cells) (Figure 1I). These results suggest that the accumulation of senescent cells and the associated inflammatory microenvironment may lead to difficulties in maintaining the regenerative homeostasis of the transplanted trachea in vivo. Therefore, targeted anti‐senescence strategies may be a potential solution for the construction of engineered tracheae.

### 2.2 Fabrication and Characterization of SF Scaffolds with Hybrid Fiber‐Film Structure

**Figure 2:**
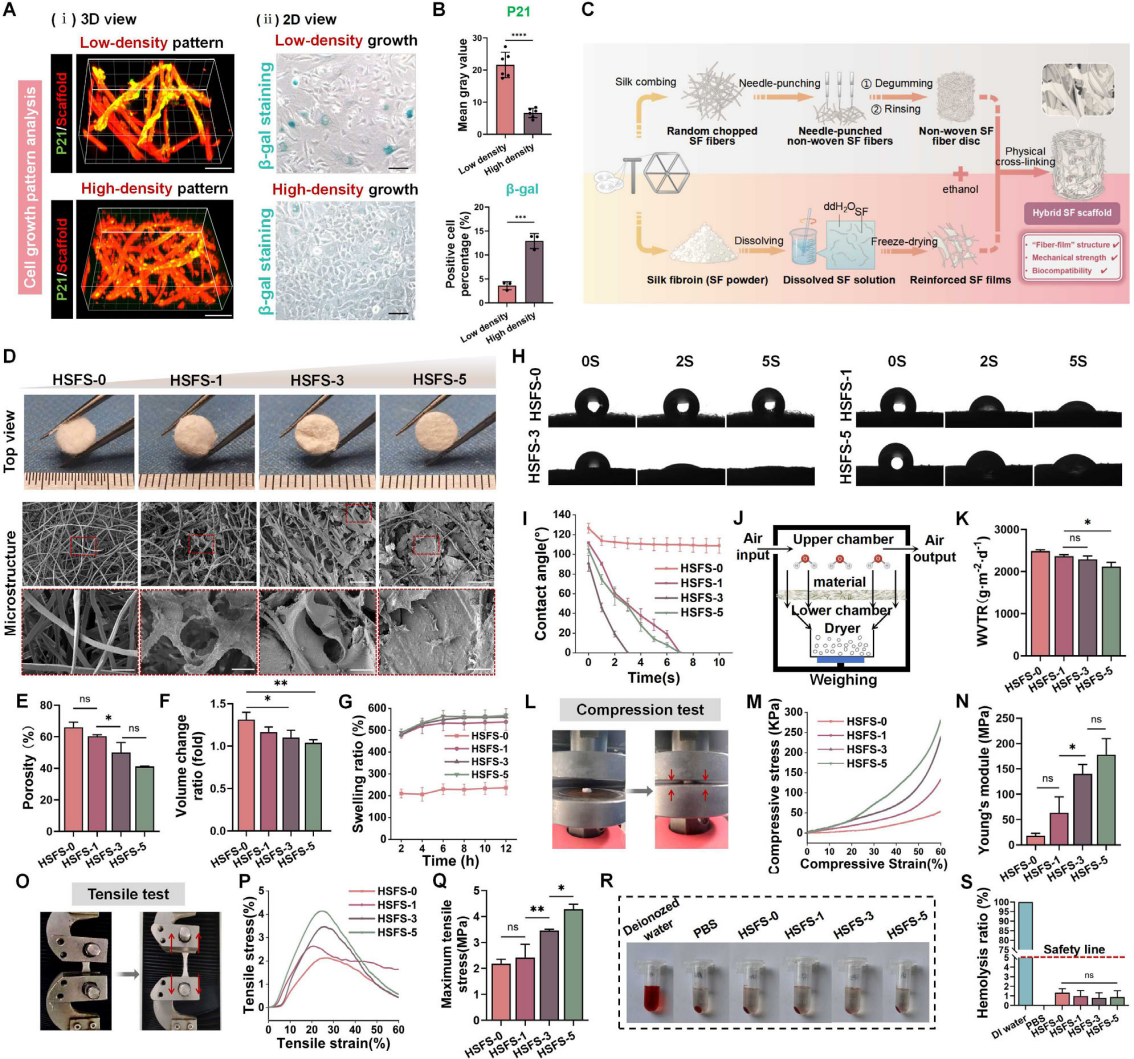
Design, manufacture, and characterization of improved HSFS with hybrid “fiber‐film” structure. A) Senescence condition with high/low‐density cell growth pattern. i) P21 IF staining of chondrocytes after inoculation on C‐SFS (three‐dimensional perspective). Scale bar: 70µm. ii) β‐gal staining of chondrocytes seeded in culture dishes (two‐dimensional perspective). Scale bar, 100µm. B) P21 fluorescence intensity (*n* = 6) and the proportion of β‐gal positive cells (*n* = 3) were quantified. C) Preparation method and process of HSFS. D) The general top view, SEM scanning image, and representative enlarged view of the scaffold with different film parameters (HSFS‐0, 1, 3, 5). Scale bar: 250 and 50µm in the large magnification view. E) Porosity calculation of HSFS, *n* = 3. F,G) Volume change and swelling curve of scaffolds in a wet environment, *n* = 3 biologically independent samples. H,I) Representative water dropping images on HSFS at 0, 2, and 5 s and the contact angle change curve within 10 s. J,K) WVT performance verification. J) Schematic diagram of the experiment. K) Quantification of WVT rate, *n* = 3. L–Q) Test of compression and tensile mechanical properties, including experimental operation diagram L,O), stress–strain curve M,P), Young's modulus N), and maximum tensile stress Q), *n* = 3. R,S) Photograph record and hemolysis rate quantification of in vitro blood compatibility test, *n* = 3. Data are presented as mean ± S.D. *N* represents biological independent samples. The *p* values were determined using one‐way ANOVA followed by Tukey's multiple comparisons test. *p < 0.05, **p < 0.01, ***p < 0.001, ****p < 0.0001.

We then performed P21 IF staining of the chondrocytes seeded on C‐SFS and found that the senescent phenotype of the cells was significantly reduced with a high‐density growth pattern. The same results were also obtained when the chondrocytes were cultured in flat Petri dishes. The proportion of β‐gal positive cells increased significantly, and the chondrocytes lost their normal polygon shape and became spindle‐like with a low‐density growth pattern, indicating the loss of the phenotype (**Figure** **2**A,B). These results suggested that increasing the contacts between chondrocytes may slow the senescence process; thus, we introduced SF film into C‐SFS to promote the development and growth of cartilage (Figure 2C).

The non‐woven fiber scaffold (HSFS‐0, namely, C‐SFS) was composed of randomly distributed isolated short fibers obtained by needle punching after silk loosing (Figure S1A, Supporting Information), which presented a filamentous appearance, and the surface became significantly denser with the introduction of more SF films (Figure 2D, Figure S1B, Supporting Information). Scanning electron microscopy (SEM) images further confirmed that the surface of the hybrid scaffold with a 1% concentration of SF film (HSFS‐1) was wrapped by the SF matrix and showed a rough state. When the concentration increased to 3% (HSFS‐3), the SF matrix film appeared on the surface of the scaffold, and the surface of the 5% SF scaffold (HSFS‐5) was covered by a larger volume of lamellar SF matrix (Figure 2D, Figure S1B, Supporting Information). As shown in Figure 2E, the addition of SF films did not change the scaffold porosity. In terms of water absorption performance, the mass of HSFS with different parameters increased by more than five times after immersion in PBS for 6 h, but the volume remained essentially unchanged (Figure 2F,G), reflecting the ability of the scaffolds to maintain their shapes in the wet environment. The results of the contact angle test showed that the droplets on the surface of HSFS‐3 disappeared the fastest (Figure 2H,I), indicating its better hydrophilicity (may be attributed to the appropriate concentration of SF film matrix, which led to the most suitable structure, which could not only play a supporting role in the scaffold, but also would not hinder cell infiltration), which exerted positive impacts on cell attachment. In addition, the water vapor transmission (WVT) experiment showed that the porosity of HSFS‐3 was not inferior to that of the HSFS‐1 group in facilitating the nutrient and oxygen permeation (*p *> 0.1), but the WVT rate of HSFS‐5 decreased significantly (*p* = 0.0119) (Figure 2J,K).

In terms of mechanical properties, the stress–strain curves reflected that both the compressive and tensile properties of the scaffolds tended to enhance with increasing SF film volume (Figure 2L,M,O,P). As for resistance to external compression, it is noteworthy that Young's modulus and compressive strain at 60% strain were not significantly different between HSFS‐3 and HSFS‐5 (*p *> 0.5), but both values were greater than those of HSFS‐1 and HSFS‐0 (Figure 2N, Figure S1C, Supporting Information). In terms of resistance to external tensile force, the maximum tensile stress of HSFS‐5 increased by nearly two folds (2.175±0.175 MPa vs. 4.282±0.19 MPa) compared with that of HSFS‐0 (Figure 2Q), which may come at the cost of loss of elastic‐plastic properties of the scaffolds, and the tensile elongation decreased as the concentration of the film increased (Figure S1D, Supporting Information). Therefore, the mechanical properties of HSFS‐3 are not inferior to those of HSFS‐5 while ensuring appropriate porosity.

Subsequently, the in vitro hemolysis test was performed to assess the blood compatibility of the scaffolds. As shown in Figure 2R, the solution of the positive control group turned red, suggesting the rupture of erythrocytes. In contrast, similar to the negative control, almost all the erythrocytes co‐cultured with the HSFS leaching solution did not rupture and showed relatively clear supernatants, indicating the good hemocompatibility of all the HSFS. Furthermore, as shown in Figure 2S, the hemolysis rates were all less than 5% (the safety standard). Finally, the blood clotting index (BCI) calculation demonstrated that the HSFS had a certain hemostatic ability, thus effectively promoting coagulation at the wound site (Figure S1E,F, Supporting Information).

### 2.3 Efficacy Assessment of HSFS to Optimize the Cartilage Growth Pattern and Promote Cartilage Development

Primary chondrocytes were cultured in HSFS leaching solution for 7 d, followed by live/dead staining and observation of the cell status under a fluorescence microscope. Results showed that the viability increased with the prolongation of the culture time, and the proportion of viable cells (green staining) was greater than 90% after 7 d, with no significant difference among all the groups (Figure S2A,B, Supporting Information). CCK‐8 assay further verified the above observations, and the chondrocytes showed high proliferative activity during the 7 d culture time (Figure S2C, Supporting Information).

**Figure 3:**
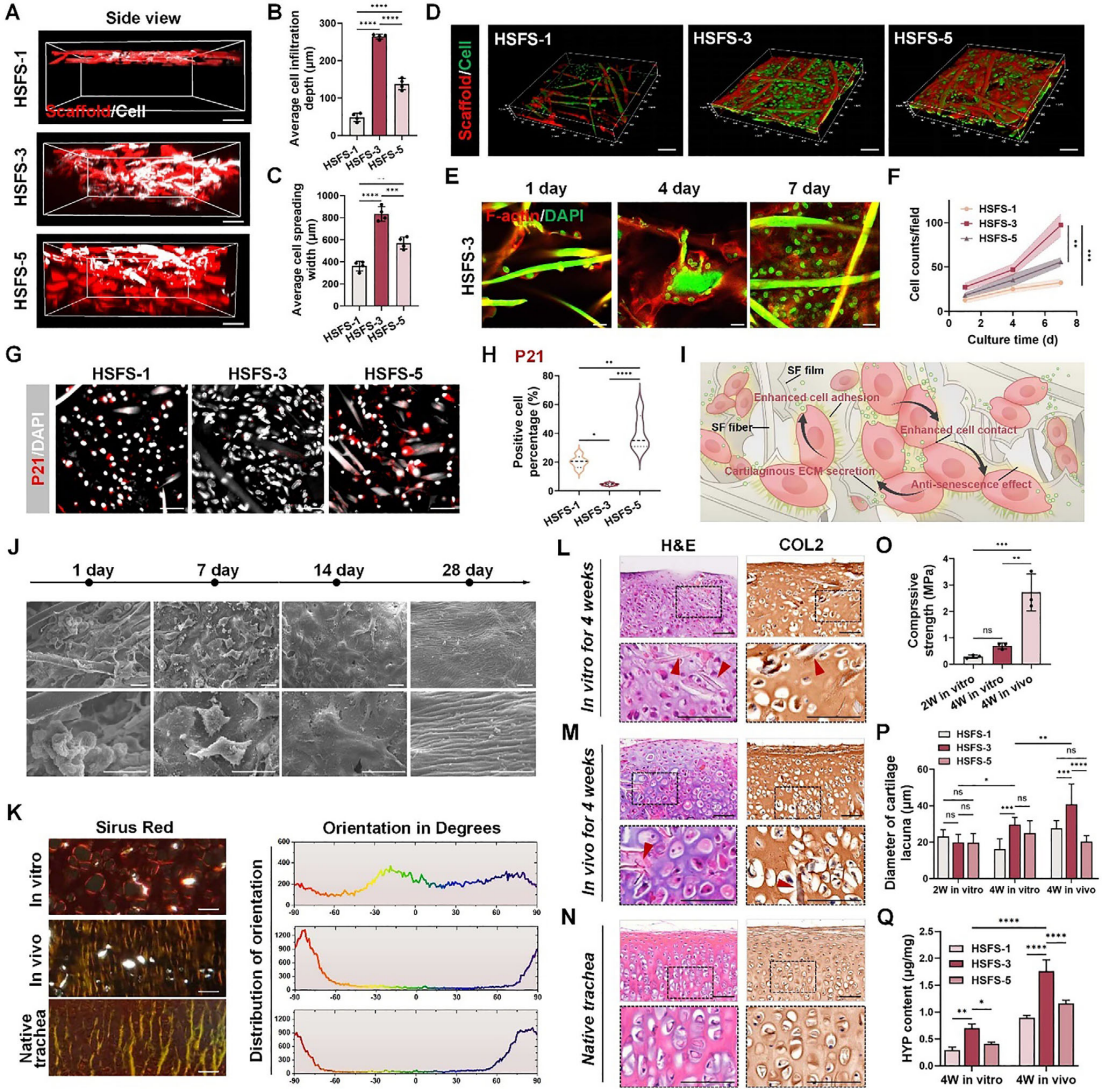
HSFS changed growth patterns and promoted the development and regeneration of cartilage tissue. A) The permeability of chondrocytes on HSFS‐1, 3, and 5 was visualized (from the side view) by F‐actin (white)/DAPI (Red) IF staining. Scale bar: 100µm. B,C) Average cell penetration depth and spreading width quantification, *n* = 4. D) Confocal 3D scanning image of the growth condition of chondrocytes 7 days after inoculation on scaffolds with different preparation parameters. Scale bar: 50µm. E,F) On the two‐dimensional plane, the representative F‐actin (red)/DAPI (green) fluorescence staining image of chondrocytes growing on HSFS‐3 for 1, 4, and 7 days E) and cell number quantification F). Scale bar: 25µm, *n*  = 3. G,H) P21 (red)/DAPI (white) fluorescent staining G) and positive cell proportion quantification H) of chondrocytes inoculated on HSFS‐1, 3, 5 for 7 days in vitro. Scale bar: 50µm, *n* = 5. I) The scheme illustrated that HSFS promoted the growth and development of cartilage by enhancing cellular contact. J) SEM images and local representative magnified views of the whole tissue 1 day, 1 week, 2 weeks, and 4 weeks after cell seeding. Scale bar: 20µm. K) Polarized light image of Sirius red staining and quantification of collagen arrangement direction for cartilage tissue (4W cultured in vitro, sequent culture for 4 weeks in Balb/c nude mice, and natural rabbit tracheal cartilage). Scale bar, 2l0µm. L–N) H&E and type II collagen immunohistochemical (IHC) staining images of cartilage cultured in vitro for 4 weeks L), continuously cultured in vivo for 4 weeks M), and natural rabbit tracheal cartilage N). The red arrow indicates the residual HSFS‐3. Scale bar: 100µm. O) The mechanical properties of cartilage tissue at different culture time points. *n* = 5. P) Diameter of cartilage lacuna, *n* = 8. Q) GAG content of cartilage tissue, *n*  = 3. Data are presented as mean ± S.D. N represents biological independent samples. The *p* values were determined using one‐way ANOVA, followed by Tukey's multiple comparisons test. *p < 0.05, **p < 0.01, ***p < 0.001, ****p < 0.0001.

To visualize the growth status of chondrocytes on HSFSs with different film parameters, IF staining for F‐actin/DAPI was performed to label the cytoskeleton after incubation for 1, 4, and 7 days, respectively. First, as shown in **Figure** **3**A–C, the side view of the scaffolds showed that cells on HSFS‐3 had the greatest penetration depth and spreading width after 7 d, whereas obviously fewer cells were retained on HSFS‐1, and the cells were strongly blocked by the 5% films and could not penetrate downward Figure S3, Supporting Information). A subsequent top view showed that chondrocytes adhered uniformly and at high density on HSFS‐3 after incubation for 7 d. We observed sparse films in HSFS‐1, with sporadic cells scattering in the fiber gaps. In contrast, the structure of HSFS‐5 was too dense, and the porosity was not enough to provide enough space for cell growth (Figure 3D). After the observation field was enlarged, we clearly observed that on the scaffold without SF films, the cells were substantially deformed by fiber stretching, but on HSFS‐3, the cells clearly aggregated and contacted each other, and the morphological homogeneity was well maintained (Figure S4A, Supporting Information). Subsequently, the 2D view (Figure 3E,F; Figure S4B, Supporting Information) revealed that although there were few chondrocytes on day 1, they all adhered to HSFS‐3 and surrounded the films to achieve mutual contact after 3 days. Cells continued to aggregate and proliferate with prolonged incubation time and finally filled the whole field of view at 7 d. In the HSFS‐1 group, the chondrocytes were mainly scattered between the pores of the scaffold, and the total number of cells was lower than that in the HSFS‐3 group. The F‐actin staining results of the HSFS‐5 group, which were consistent with those of the 3D view, showed that the films were too dense for the cells to penetrate and contact each other in the scaffold. Under different growth patterns, the number of P21‐positive chondrocytes on HSFS‐3 was significantly reduced (Figure 3G,H), which demonstrated that cell–cell contact and communication inhibited cell senescence and helped maintain the original chondrocyte phenotype (Figure 3I).

HSFS‐3 has good mechanical properties as well as suitable porosity, which is favorable for chondrocyte infiltration, adhesion, and close‐packed cellular contact. Therefore, we chose this parameter for subsequent cartilage tissue construction, developed cartilage tissue in vitro using induction medium, and implanted this tissue subcutaneously into nude mice for further evaluation of the regenerative capability in vivo.

First, the microstructure of the chondrocyte tissue was determined by SEM during 4W culture in vitro. Initially, the chondrocytes adhered to the scaffold, and then, the cells proliferated and covered the scaffold surface. After 2 weeks, the cells began to merge and secrete matrix, and finally, the cells could not be observed, with the scaffold surface completely wrapped by abundant cartilaginous matrix (Figure 3J). In addition, with prolonged in vitro culture time, the surface of the cartilage tissue became dense. After 4 weeks of culture in vitro, the cartilage ring had great elasticity and mechanical strength (Movie. S1, Supporting Information). After subcutaneous embedding in nude mice for 4 weeks, the color of the cartilage tissue changed from yellow to ivory white, suggesting that the cartilage tissue had matured after the in vivo development process (Figure S5A–C; Movie. S2, Supporting Information). This conclusion was also verified by the further reduction in P21^+^ senescent cells in the cartilage after being embedded in vivo (Figure S7, Supporting Information).

Type II collagen (Col II) in natural cartilage is distributed in a network and plays a key role in maintaining mechanical strength. We performed Sirius red staining and observed the direction arrangement of the Col II network under the polarizing microscope in the cartilage tissues at each culture time point. Results showed that the density of cartilaginous extracellular matrix (ECM) increased significantly after implantation in vivo, and the collagen fibers inside the tissues intersected with a network structure, similar to that of natural tracheae (Figure 3K).

Finally, histological staining was performed to further confirm that, after 4 weeks of in vivo culture in nude mice, the deposition of cartilage‐specific ECM increased significantly, resembling that of natural tracheae (Figure 3L–N), and the anti‐compressive strength of the tissues increased (Figure 3O). Compared with those of in vitro cultured cartilage, the number and size of cartilage lacunae were greater, demonstrating that the cartilage tissue was well developed and had become mature (Figure 3P, Figure S6D, Supporting Information). In addition, the levels of cartilage‐related biochemical indices (including the DNA content, glycosaminoglycan (GAG) content, and hydroxyproline (HYP) content) gradually increased with increasing in vitro culture time (Figure 3Q, Figure S6E,F, Supporting Information). Notably, we still inoculated and cultured cells at the same density on scaffolds with different parameters (HSFS‐1 and HSFS‐5). The results of histological staining and cartilage‐related biochemical analyses showed that cartilage regeneration was not as effective as that in the HSFS‐3 group (Figure S6, Supporting Information).

### 2.4 Immune Niche Regulation by Oxidative Stress Modulation and Mitochondrial Protection by Quercetin

**Figure 4:**
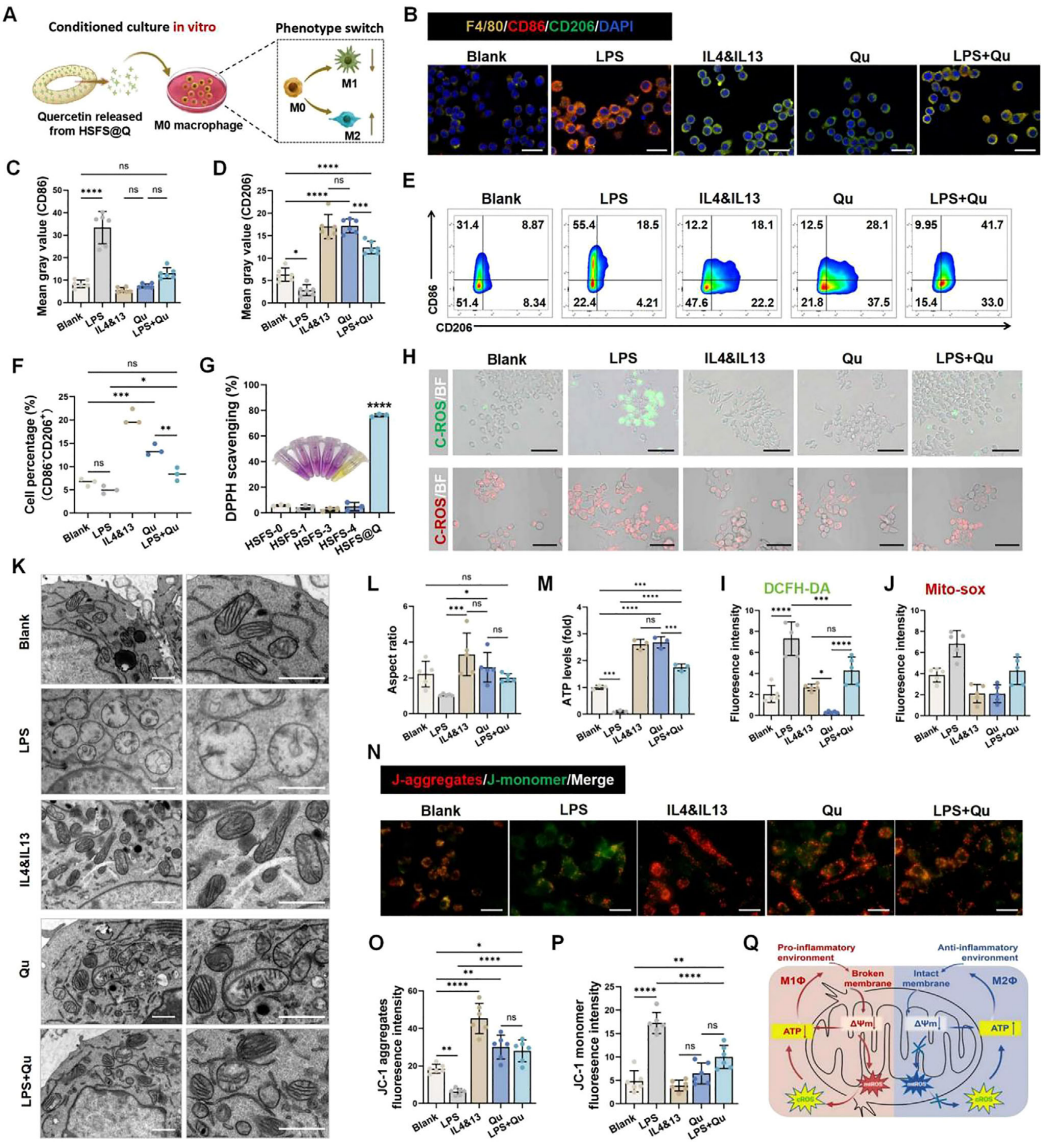
The regulatory impacts of quercetin on macrophages in vitro and the mechanism exploration. A) Schematic diagram of quercetin regulating macrophage polarization. B–F) Macrophages were cultured for 4 days under different conditions (in complete culture and supplemented with LPS, IL4&13, quercetin, and quercetin+LPS). B) IF staining: F4/80 (yellow), CD86 (red), CD206 (green), and nucleus (DAPI, blue), scale bar: 25µm. C,D) Quantitative analysis of fluorescence intensity of CD86 and CD206, *n* = 6. E) CD86 and CD206 representative flow cytometry scatter diagram. F) CD86‐/CD206+ cell ratio in flow cytometry analysis, *n* = 3. G) DPPH radical scavenging capacity of the scaffold's leaching solution. H–J) Detection of intracellular and mitochondrial ROS in macrophages. H) Under different culture conditions, macrophages were stained with DCFH (green) and MitoSOX (red) kits. Scale bars: 100 and 50µm, respectively. I,J) DCFH and MitoSOX fluorescence intensity quantification, *n* = 5. K) Transmission electron microscope (TEM) scanning images and a representative magnified view of mitochondria in macrophages. Scale bar: 1µm. L) Morphological analysis of mitochondria in K, aspect ratio referred to the ratio of long and short axes, *n* = 5. M) ATP production level of macrophages, *n* = 3. N–P) JC‐1 fluorescence staining for macrophage mitochondrial membrane potential, JC‐1 monomer (green), JC‐1 aggregate (red), and fluorescence intensity quantification under different treatment conditions. Scale bar: 30µm. *n* = 6. Q) Mechanism diagram of quercetin's immune regulation (macrophage reprogramming). *N* represents biological independent samples. The *p* values were determined using one‐way ANOVA followed by Tukey's multiple comparisons test. *p < 0.05, **p < 0.01, ***p < 0.001, ****p < 0.0001.

Immune regulation through macrophage reprogramming is the second way to resist senescence. To simulate the inflammatory environment in vivo, we used lipopolysaccharide (LPS)‐induced M1‐phenotype macrophages as the negative control (LPS group), IL4&IL13‐induced M2‐phenotype macrophages as the positive control (IL4&IL13 group), and M0‐phenotype macrophages cultured in complete medium as the blank group (**Figure** **4**A).

Cell morphology and specific markers are often used to distinguish macrophages with different phenotypes.^[^ ^23^ ^]^ Light microscopy and F‐actin fluorescence staining revealed a greater degree of deformation and more filamentous pseudopods of M1‐phenotype macrophages in the LPS group. On the contrary, macrophages cultured with the leaching solution from HSFS‐3 loaded with quercetin (HSFS@Q) (Qu group) presented a flatter shape with lamellar feet (the typical characteristic of M2‐phenotype macrophages), suggesting that quercetin‐impacted macrophages tended to convert to the M2 phenotype (Figure S8A,B, Supporting Information). CD86 and CD206 are specific markers for M1 and M2‐phenotype macrophages, respectively. The CD206 fluorescence intensity was greater in the Qu group than in the other groups and was not significantly different from that in the IL4&IL13 group. After quercetin was added to the inflammatory environment (LPS+Qu group), CD206 expression exceeded that of the blank group (*p*<0.0001), which further confirmed the efficacy of quercetin in inducing M2‐phenotype macrophage polarization (Figure 4B–D, Figure S8C, Supporting Information). The flow cytometric results also showed that more macrophages in the Qu group expressed CD206, which was maintained under LPS stimulation (Figure 4E,F, Supporting Information).

Recent research advances have suggested the critical role of the immuno‐metabolic profile in the activation and associated functional changes of macrophages.^[^ ^24^ ^]^ The polarization of macrophages may be determined by mitochondrial function and metabolic cascade responses, such as reactive oxygen species (ROS) homeostasis.^[^ ^25^, ^26^ ^]^ We therefore explored the effect of quercetin on macrophage mitochondrial function, and the groups were divided as described above. First, as shown in Figure 4G, the antioxidant activity (DPPH scavenging capacity) of the scaffolds was significantly increased after quercetin loading, reaching 76.18 ± 1.131%, and there were no significant changes in other material properties (including structure, swelling capacity, mechanical properties, and biocompatibility) (Figure S9, Supporting Information). HSFS itself had no antioxidant capacity, and there were no significant differences among the different preparation parameters (Figure 4G).

Then cytology experiments were carried out to determine the total cellular ROS and mitochondrial superoxide production levels in macrophages with the DCFH‐DA probe and MitoSOX reagents, respectively. Almost no DCFH‐DA positive cells were found in the Qu group, and the intensity of superoxide in the mitochondria was also significantly reduced (Figure 4H,J). Then, we observed the effect of quercetin on the mitochondrial micromorphology of macrophages. Under inflammatory conditions (LPS group), many swollen mitochondria, characterized by the disappearance of mitochondrial cristae, appeared in the macrophages. After quercetin treatment, the structure recovered and approached the normal shape of the positive control group (Figure 4K,L), and ATP was synthesized at the normal level (Figure 4M). The accumulation of ROS, including superoxide, reduces the mitochondrial membrane potential (ΔΨm), leading to mitochondrial membrane rupture. The JC‐1 fluorescence probe showed that the intensity of JC‐1 monomers in macrophages significantly increased after quercetin treatment, indicating an increase in the ΔΨm (Figure 4N–P), thus ensuring the stability of the mitochondrial structure.

The above results suggested that the application of quercetin could exert the anti‐oxidative stress effect through mitochondrial empowerment and ROS scavenging, thus achieving protective effects on mitochondria to induce M2‐phenotype macrophage polarization, which regulated the immune microenvironment in the anti‐inflammatory direction (Figure 4Q).

### 2.5 In Vitro Validation that Quercetin Promotes Multiple Tissue Regeneration through an Immune‐Senescence Cascade

**Figure 5:**
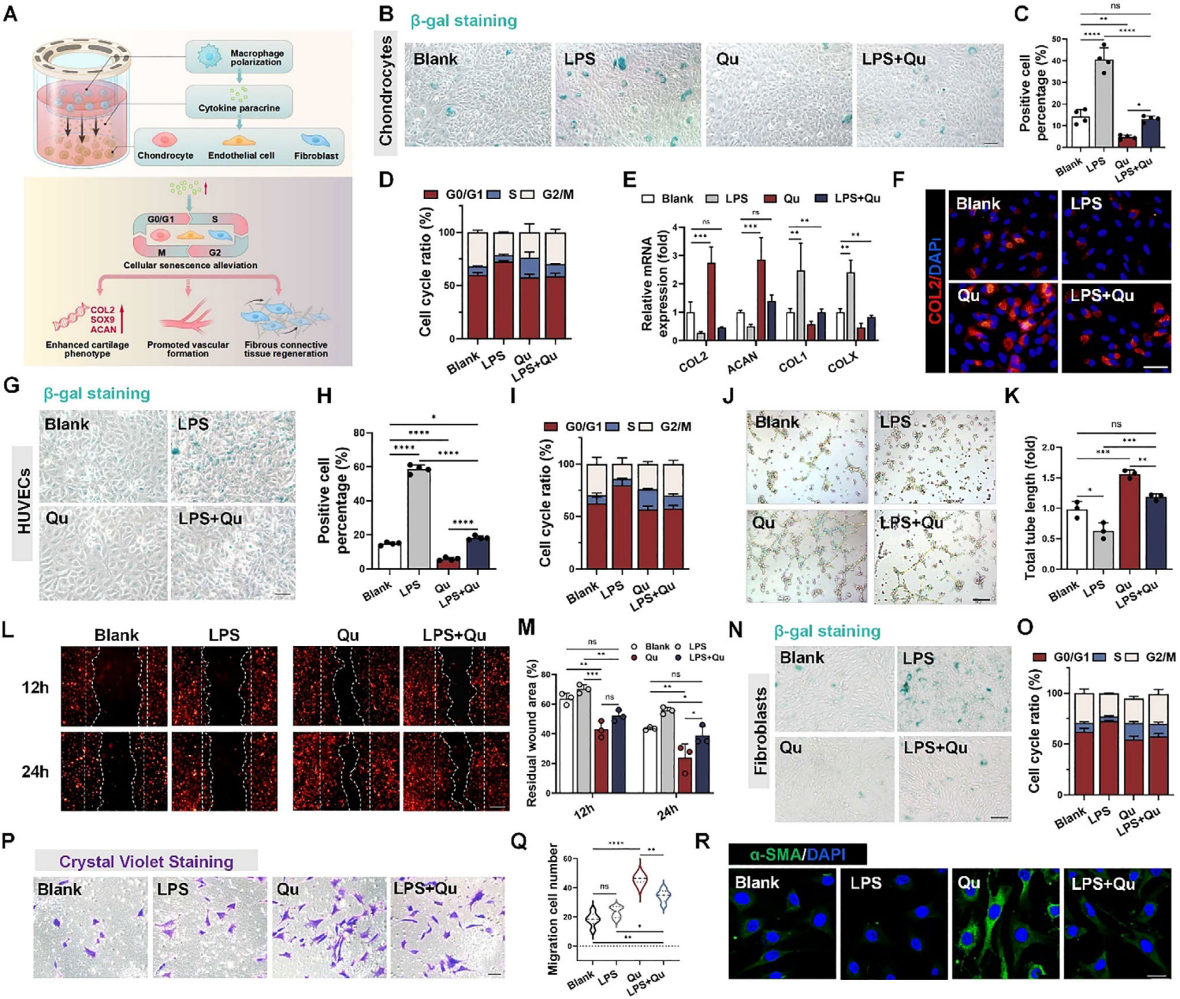
In vitro validation of quercetin promoting multiple cell lineage regeneration through the immune‐senescence cascade. A) Schematic diagram of the cellular co‐culture system and functional verification. B–F) Related changes of rabbit primary chondrocytes under different culture conditions in vitro. B,C) β‐gal staining of chondrocytes and quantification of the proportion of positive cells. Scale bar: 100µm. *n* = 4. D) Cell cycle analysis of chondrocytes. *n* = 3. E) The expression of cartilage‐related genes (COL2, ACAN, COL1, and COLX), *n* = 3. F) COL2 IF staining (red) of chondrocytes under different culture conditions. Scale bar: 100µm. G–M) Related changes of human umbilical vein endothelial cells (HUVECs) under different culture conditions in vitro. G,H) β‐gal staining of HUVECs and quantification of the proportion of positive cells. Scale bar, 100µm. *n* = 4. I) Cell cycle analysis of HUVECs, *n* = 3. J) The typical images of HUVEC tube formation. Scale bar: 200µm. K) Quantitative analysis of total tube length in (J). *n* = 3. L) Representative migration images of HUVECs labeled with cell tracker Dil (red). Scale bar: 200µm. M) Quantitative analysis of HUVECs migration, *n* = 3. N–R) Related changes of 3T3 fibroblasts under different culture conditions in vitro. N) β‐gal staining of fibroblasts. Scale bar: 100µm. O) Cell cycle analysis of fibroblasts, *n* = 3. P,Q) Crystal violet staining and migration cell count of fibroblasts under different culture conditions. Scale bar: 50µm. *n* = 6. R) α‐smooth muscle actin (α‐SMA) IF staining (green) of fibroblasts under different culture conditions. Scale bar: 25µm. *N* represents biological independent samples. The *p* values were determined using one‐way or two‐way ANOVA followed by Tukey's multiple comparisons test. *p < 0.05, **p < 0.01, ***p < 0.001, ****p < 0.0001.

To mimic the in vivo microenvironment as much as possible, we established a Transwell co‐culture system of macrophages with chondrocytes, endothelial cells, and fibroblasts to reveal the potential of quercetin to slow the senescence of the three lineages of cells by immune microenvironment regulation, thus promoting the regeneration of multiple tracheal components (cartilage, blood vessels, and fibrous connective tissues) (**Figure** **5**A).

According to the results of β‐gal staining, γH2AX and high mobility group box‐1 protein (HMGB1) immunofluorescence staining (Figure S10A–D, Supporting Information), growth factors (including IGF‐1, FGF‐2, and TGF‐β1) secreted by anti‐inflammatory macrophages induced by quercetin (Qu group) (Figure S10E–G, Supporting Information) slowed the senescence process of chondrocytes by preventing DNA damage and inflammation spreading, which promoted entry into the proliferative phase of the cell cycle and alleviated the G1 phase arrest of the cell cycle when cells stopped dividing in the inflammatory environment (LPS+Qu group vs. LPS group: 11.533±0.3479% vs. 5.663±0.784%) (Figure 5B–D). In addition, qPCR showed that in the microenvironment regulated by quercetin, the expression of cartilage function‐related genes such as COL2 and ACAN increased significantly (*p *< 0.001), and the expression of genes related to cartilage dedifferentiation and hypertrophy, such as COL1 and COLX decreased (Figure 5E). Moreover, toluidine blue and COL2 staining were performed, which further proved that the addition of quercetin promoted chondrogenic‐specific matrix secretion in the inflammatory environment (Figure 5F, Figure S10f–H, Supporting Information). The above data indicate that quercetin stimulates M2‐phenotype macrophage polarization, which could help reshape the immune microenvironment, in which chondrocytes could consolidate and promote the cartilage phenotype by delaying senescence.

We also observed the growth and development of endothelial cells and fibroblasts in the co‐culture system. Similarly, β‐gal staining, γH2AX, and HMGB1 immunofluorescence staining showed that the senescence and cell cycle arrest of endothelial cells and fibroblasts could be alleviated by immune microenvironment remodeling (Figure 5G–I and N–O; Figures S11A–D and S12A–E, Supporting Information). Enzyme‐linked immunosorbent assay (ELISA) revealed that anti‐inflammatory macrophages induced by quercetin culture (Qu group) secreted higher concentrations of the angiogenic factors TGF‐β3, VEGF‐A, and PDGF‐AA (Figure S11E–G, Supporting Information). The expression of angiogenesis‐related genes (VEGF, CD31, and HIF‐1α) was significantly increased in endothelial cells (*p *< 0.001) (Figure S11H, Supporting Information). In the tube formation assay, it can be seen under the light microscope that more vascular junctions formed and that the total tube length was longer in the Qu group than in the other groups. However, in contrast, the vascular network in the LPS group was difficult to form (Figure 5J,K, Figure S11I, Supporting Information). Subsequently, the results of the scratch test and crystal violet staining showed that the migration of endothelial cells and fibroblasts in the LPS+Qu group was significantly restored compared with that in the LPS group (Figure 5L,M,P,Q), suggesting the potential for regeneration of fibrous connective tissue and blood vessels therein. In addition, fibroblasts were stained with α‐SMA, a specific marker of fibrous tissue, and the fluorescence intensity in the Qu group significantly increased (Figure 5R; Figure S12F, Supporting Information). These data indicate that endothelial cells and fibroblasts could respond to the anti‐inflammatory effect of quercetin to maintain a stable state and restore the mature phenotype.

### 2.6 Further Functional Maintenance and Development of Multiple Tracheal Tissues In Vivo

**Figure 6:**
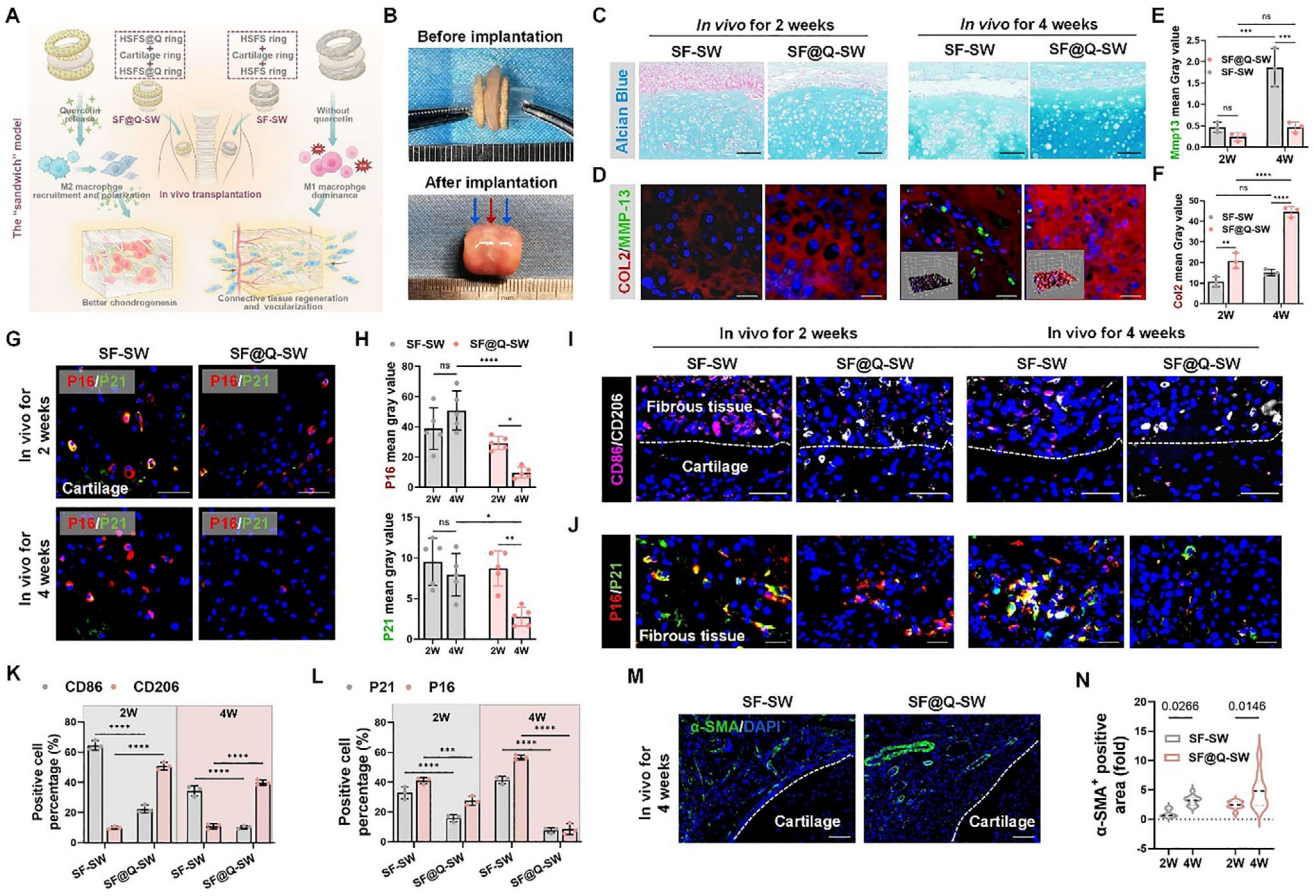
Construction of the sandwich model and validation of tracheal multiple tissue regeneration in vivo. A) Schematic diagram of the sandwich model and tissue regeneration verification. B) Gross photographs of the sandwich model before and after 4 weeks of intramuscular embedding. The red arrow represents cartilage; The blue arrow represents regenerated fibrous connective tissue. C) Alcian blue staining of sandwich tissues (SF‐SW and SF@Q‐SW) after embedding (including the fibrous connective tissue above and the cartilage below). Scale bar: 100µm. D) COL2 (red) and MMP13 (green) IF staining and 3D surface plot analysis of fluorescence intensity. Scale bar: 25µm. E,F) Quantitative analysis of fluorescence intensity of COL2 and MMP13 in (D). *n* = 3. G,H) P16 (red) and P21 (green) IF staining and fluorescence intensity quantification of cartilage in the sandwich model. Scale bar: 50µm. *n* = 5. I,J,M) If staining for the inflammatory indicators (CD86, CD206), senescence indicators (P16, P21), and mature vascular indicators (α‐SMA) in the fibrous connective tissue of the sandwich model. Scale bar: 20, 50, and 100µm, respectively. K,L) Quantification of the proportion of positive cells based on (I,J). *n* = 3. N) Positive area analysis based on (M). *n* = 9. N represents biological independent samples. The *p* values were determined using two‐way ANOVA followed by Tukey's multiple comparisons test. *p < 0.05, **p < 0.01, ***p < 0.001, ****p < 0.0001.

In order to explore the regeneration of multiple tracheal tissues in vivo, we first constructed the “sandwich” model (**Figure** **6**A). First, after 4 weeks of muscle embedding in the rabbit neck, an obvious three‐layer structure was observed in the “sandwich” model loaded with quercetin (SF@Q‐SW), indicating that the overall regenerated tissue was in a good state and that the structure remained stable (Figure 6B). Next, we evaluated the development of cartilage tissue in vivo, and found that compared with that at 2 weeks, obviously more cartilage matrix was retained after 4 weeks of SF@Q‐SW embedding, and the cartilage lacunae showed greater maturity without degradation. However, without quercetin loading, the cartilage tissue of the SF‐SW group significantly degraded after 4 weeks in vivo, and more matrix metalloproteinase (MMP13)‐positive cells appeared inside the tissue (Figure 6C–F; Figure S13A, Supporting Information). Then, fluorescence staining of P16 and P21 was performed on the cartilage tissue of the SF@Q‐SW and SF‐SW groups, and we found that in the former group, there were fewer senescent chondrocytes, whereas without quercetin, it was difficult to prevent senescence (Figure 6G,H).

Subsequently, we evaluated the regeneration of fibrous connective tissue. First, the fibrous connective tissue of the SF@Q‐SW group contained more M2‐polarized macrophages (Figure 6I,K), and the degree of tissue oxidative stress and senescence was significantly reduced through immune regulation (Figure 6JL; Figure S14A, Supporting Information). After 4 weeks, there were almost no P16 or P21 positive cells (less than 10%), indicating the occurrence of anti‐inflammatory and anti‐senescence cascades. However, if quercetin was not added, senescent cells could not be eliminated, and the number of senescent cells tended to increase after 4 weeks (Figure 6L). In addition, α‐SMA staining of the sandwich model showed that the fibrous connective tissue regenerated well and that there was more mature vascular regeneration inside (Figure 6M,N).

Subsequently, we also constructed the subcutaneous embedding model to evaluate the regeneration of fibrous connective tissue. Figure S15A (Supporting Information) shows the H&E staining of peripheral connective and muscle tissues after the scaffolds were subcutaneously embedded in rats. We found that the subcutaneous tissues gradually infiltrated the quercetin‐loaded scaffolds (SF@Q‐S) as the embedding time increased. The non‐quercetin‐loaded scaffold (SF‐S) induced an acute and severe inflammatory response, and inflammatory cells gradually invaded the scaffold from the surrounding tissues, forming an inflammatory hyperplasia layer. In contrast, when the scaffold was loaded with quercetin and implanted for 4 weeks, the depth of subcutaneous tissue infiltration was significantly greater than that of the inflammatory hyperplasia layer (Figure S15A,B, Supporting Information). In addition to the ability to recruit peripheral tissue infiltration, another key point was to evaluate the ability of SF@Q‐S to induce vascular reconstruction in the infiltrated tissue. CD31 IF and immunohistochemical (IHC) staining showed that the subcutaneous vessels in the SF@Q‐S group tended to transform into mature blood vessels with larger lumens after 4 weeks (Figure S15C,D, Supporting Information), which was consistent with the results of the sandwich model.

In addition, after subcutaneous embedding for 4 weeks, the acute inflammatory reaction in the infiltrating tissue inside the scaffold was downregulated, and the proportion of neutrophils was reduced (Figure S16A, Supporting Information). CD86 and CD206 IF staining combined with quantitative analysis showed that no matter how long the embedding time was, there were more pro‐inflammatory M1 macrophages around SF‐S, while SF@Q‐S could recruit more M2‐type macrophages for tissue repair and regeneration (Figure S16B,C, Supporting Information). These results were consistent with that in the sandwich model.

In conclusion, the above results indicated that the regeneration of multiple components (cartilage, fibrous connective tissue, and blood vessels) of the trachea could be further enhanced in vivo. According to the comparison results with the control group, it was mainly due to the quercetin‐mediated immune‐senescence cascade, which restored cell viability and removed senescent cells to achieve further tissue development in vivo.

### 2.7 Orthotopic Transplantation of the Hebe Engineered Trachea into Rabbits with Segmental Tracheal Defects

**Figure 7:**
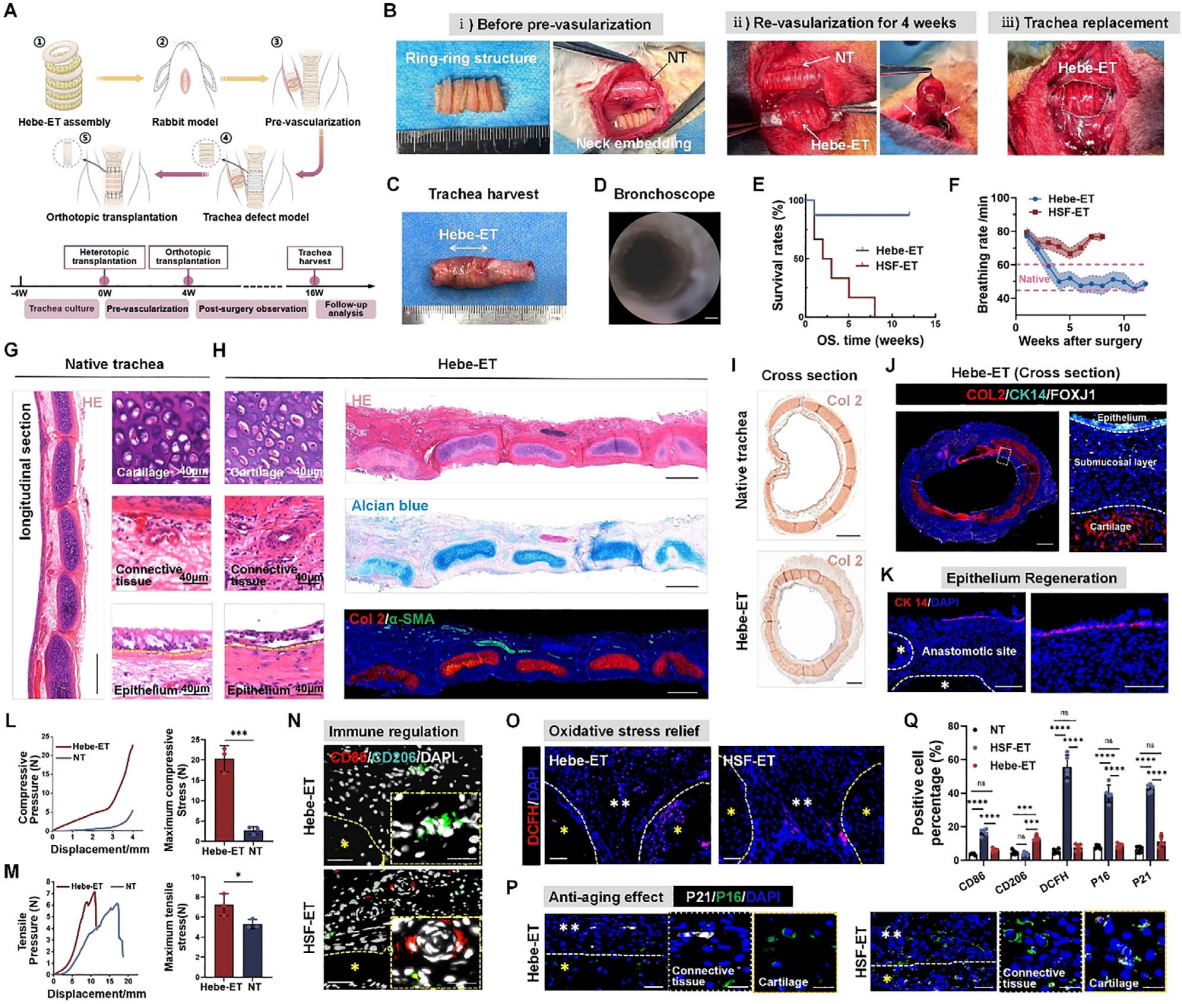
Orthotopic transplantation of the engineered trachea. A) Schematic diagram of orthotopic transplantation. B) I) The Hebe‐ET was assembled and ectopically embedded for pre‐vascularization; II) The pedicled Hebe‐ET after 4‐week pre‐vascularization. The white arrow indicated the vascular pedicle; III) End‐to‐end anastomosis between the Hebe‐ET and the natural rabbit trachea (NT). The Hebe‐ET was outlined by the dotted line. C) Photographs of the Hebe‐ET sampled 12 weeks after transplantation. D) Bronchoscope images. Scale bar: 1mm. E) Survival curve of experimental rabbits in Hebe‐ET group, and HSF‐ET group as control, *n* = 8 and 5, respectively. F) Breathing monitoring of rabbits. G,H) H&E and Alcian blue staining, COL2, and α‐SMA IF staining for cartilage, fibrous connective tissue, and epithelium of NT and Hebe‐ET (longitudinal sections). Scale bar: 500µm (NT) and 1mm (Hebe‐ET). I) COL2 IHC staining of representative cross sections of NT and Hebe‐ET. Scale bar: 1mm. J) COL2 (red)/CK14 (green)/FOXJ1 (white) IF staining of Hebe‐ET. Scale bar: 1mm (low magnification view) and 100µm (high magnification view). K) CK14 fluorescence staining images of epithelium at the anastomosis site of Hebe‐ET (longitudinal section). The white asterisk represented the cartilage. Scale bar: 100µm (low magnification view) and 50µm (high magnification view). L,M) The representative compressive and tensile stress‐displacement curve, as well as the maximum compressive and tensile stress of Hebe‐ET and NT. *n* = 3. N–P) The immune regulation, oxidative stress relief, and anti‐senescence properties of Hebe‐ET. N. CD86 (red) and CD206 (green) fluorescence staining. The single yellow asterisk represented cartilage. Scale bar: 50µm (low magnification view) and 25µm (high magnification view). O) DCFH fluorescence staining, the white dotted line represents the boundary between cartilage and fibrous connective tissue. The double white asterisk represented connective tissue, and the single yellow asterisk represented cartilage. Scale bar: 50µm. P) P21 and P16 fluorescence staining. The white dotted line and asterisk represented the same as (O). Scale bar: 50µm (low magnification view) and 25µm (high magnification view). Q) Positive cell proportion of each marker in (M–P). *n* = 5. N represents biological independent samples. The *p* values were determined using a one‐tailed unpaired t‐test. *p < 0.05, **p < 0.01, ***p < 0.001, ****p < 0.0001.

We constructed a tracheal‐like structure with alternate stacking of cartilage and fibrous connective tissue rings. First, The Hebe‐ET was ectopically embedded into the para‐tracheal muscles of rabbits for 4 weeks to initiate pre‐vascularization, and the vascular pedicles surrounded the trachea with good blood supply. The vascularized bioengineered trachea was subsequently transplanted into the tracheal defect for reconstruction (**Figure** **7**A,B). Gross and bronchoscopy images showed that Hebe‐ET was similar to the natural trachea, with great tensile properties, an obvious “ring‐ring” structure, smooth lumen walls, and no signs of stenosis 12 weeks after surgery (Figure 7C,D; Movie. S3, Supporting Information). A total of 87.5% of the animals survived after 12 weeks, breathed stably, and maintained a normal respiratory rate (Figure 7E,F).

Histological staining of tracheal longitudinal sections showed that the structure of Hebe‐ET was perfectly similar to that of the natural trachea. The ring–ring cartilage structure of the tracheal substitute was well preserved and showed typical lacunae. The overall structure of the connective tissue was similar to that of the natural trachea, which showed good regenerative conditions with functional vascular regeneration. The inner wall of Hebe‐ET was covered by the epithelium (Figure 7G,H; Figure S17A, Supporting Information). Thus, regeneration of the trachea in the whole layer (cartilage, connective tissue, and epithelium) was realized via Hebe‐ET. In addition, H&E and COL2 IHC staining of the cross sections of Hebe‐ET further demonstrated that the entire cartilage rings were homogenized after long‐term transplantation, without fracture or degradation in a certain area (Figure 7I; Figure S17B, Supporting Information). In contrast to Hebe‐ET, after the engineered trachea without quercetin loading (HSF‐ET) was transplanted in vivo for 4 weeks, the “ring–ring” structure was no longer obvious in general, the lumen got narrowed, and the fibrous tissue infiltrated into the cartilage, which was almost completely degraded (Figure S18, Supporting Information). In addition, with regard to epithelium regeneration, it is worth mentioning that H&E and CK14/FOXJ1 fluorescence staining of the longitudinal and cross tissue sections at the anastomotic site suggested that the epithelium of the natural trachea migrated to the engineered trachea (Figure 7J,K; Figure S17B, Supporting Information). Furthermore, the mechanical strength (compressive and tensile strength) of the transplanted tracheal substitute even exceeded that of the natural rabbit trachea (Figure 7L,M, Supporting Information).

Finally, IF staining for CD86 and CD206 showed the presence of M2‐phenotype rather than M1‐phenotype macrophages in the fibrous connective tissue of the Hebe‐ET group compared with those in the control group, and the level of oxidative stress was significantly reduced in this region (Figure 7N,O). Senescence‐related staining verified that all the components of Hebe‐ET were in youth with no signs of senescence. However, the regenerative microenvironment was disturbed without quercetin, leading to senescence and consequent functional loss of HSF‐ET after transplantation (Figure 7P,Q).

## 3 Discussion

There is still a lack of clinical solutions for the repair of segmental tracheal defects. Previous reports have investigated tracheal reconstruction with allogenic and autologous tissue transplantation, but major clinical limitations still exist, such as insufficient blood supply and necrosis of the graft, tracheomalacia, and stenosis, and the need for patients to take immunosuppressants for a long time.^[^ ^27^, ^28^, ^29^ ^]^ With the application of material engineering and manufacturing in biomedicine, tissue engineering has provided new ideas for the construction of tracheal substitutes.^[^ ^30^ ^]^ They can be implanted in vivo to effectively replace or rebuild the normal structure and function of damaged tissues after mimicking the cellular composition and tissue structure of natural tissues in vitro. In recent years, researchers have been designing and developing biomimetic tracheal substitutes. Neville et al.^[^ ^8^ ^]^ were the first to propose an enclosed tubular prosthesis made of silicone to construct an artificial trachea. However, due to the lack of biocompatibility, complications such as infection and granulation hyperplasia occurred after implantation, leading to restenosis of the airway and poor patient prognosis. Subsequently, scholars have attempted to construct biocompatible and functional tracheal substitutes. Representative studies, such as those by Xu and Sun et al.^[^ ^31^, ^32^ ^]^, initially realized simple tracheal functional reconstruction by mimicking the anatomical “ring–ring” structure, but reports on the long‐term maintenance of the multi‐component function of engineered tracheae (ET) after transplantation, which is the key to determining its clinical applicability, are lacking.^[^ ^31^, ^32^, ^33^ ^]^ Therefore, this work aimed to construct an engineered trachea with post‐transplanted regenerative homeostasis.

However, the causes of ET failure in vivo remain unknown, so the theoretical basis of tracheal design is still limited; thus, realizing a breakthrough in therapeutic efficacy is difficult. This work focused on overall tracheal senescence as the driving factor of dysfunction. Senescence is associated with a wide range of clinically important degenerative diseases.^[^ ^3^, ^4^ ^]^ However, this topic has not received much attention in the field of tracheal regeneration. As a result, this study proposed, for the first time in this field, dual anti‐senescence designs for the maintenance of post‐transplanted ET functions.

First, if the cartilage tissue develops poorly in vivo, accompanied by the degradation of the biomaterial loaded with chondrocytes, the original secreted chondrogenic ECM dissipates, and the mechanical properties of the trachea rapidly weaken, leading to the collapse of the tracheal lumen.^[^ ^34^, ^35^ ^]^ Therefore, factors such as signal communication between materials and cells, mechanical guidance to cells, and their relative spatial distribution not only guide cellular growth but also determine the fate of regenerative tissue. As the carrier, the stiffness, viscoelasticity, ligand density, and pore size of the hydrogel matrix affect its properties. In particular, the chemical bonds inside the hydrogel restrict cells in all directions and cut off the contact between cells, easily leading to a decline in cell vitality and loss of phenotype.^[^ ^11^, ^36^, ^37^ ^]^ Therefore, in this study, we chose to manufacture effective functional scaffolds for cartilage regeneration and accurately replicate the intrinsic characteristics of natural cartilage. The material composition, structure, and related mechanical properties of the scaffolds were carefully considered and realistically designed. In terms of material selection, SF extracted from silk cocoons was chosen because of its clinical translation potential.^[^ ^9^, ^10^ ^]^ However, for the application of SF scaffolds in cartilage regeneration, the following problems need to be solved: 1) sufficient mechanical support; 2) the ability to facilitate cell infiltration and retention; and 3) the ability to provide biochemical clues for cell growth and development. At present, SF scaffolds are prepared mainly by electrospinning or 3D printing, or by freeze‐drying or gas foaming.^[^ ^38^, ^39^, ^40^ ^]^ However, in the previous case, SF microfibers in dense arrangements often form a compacted scaffold structure, which is not conducive to chondrocyte infiltration and thus hinders the regeneration of bulk tissues. In the latter case, the SF scaffold has too large pores, resulting in poor mechanical strength, and the cells are difficult to retain. In this study, we designed a special “fiber‐film” structural SF scaffold, which was superior to a single component in terms of reinforcing performance through synergistic effects. The non‐woven SF short fibers were prepared by the textile needle punching method, and then reinforced SF films were introduced and cross‐linked with ethanol to form a unique hybrid fiber‐film scaffold., in which non‐woven fibers could form anisotropic inter‐connected pores, making it easier for chondrocytes to penetrate the whole scaffold, which was verified in our 3D visualization images. Furthermore, SF molecules in reinforced films can tightly combine with SF fibers through protein secondary structure rearrangement (predominantly β‐pleated sheets) and self‐assembly after cross‐linking, thus providing additional mechanical support for the scaffold.^[^ ^41^, ^42^ ^]^ More importantly, as a hydrophilic matrix, the surface of the SF films provided a great many adhesion sites for the proliferation of chondrocytes, improved cell retention and overall spatial distribution, and facilitated three‐dimensional connections and communication between cells, thus increasing cell vitality, slowing senescence, ultimately promoting cell development and cartilaginous matrix deposition, and obtaining mechanically strengthened cartilage tissue, which could further develop in vivo and not degrade after orthotopic transplantation.

Deterioration of the regenerative microenvironment is a key factor in cellular senescence, with impacts including clonal expansion and infiltration of immune cells in regenerative niches and loss of functional cellular phenotypes.^[^ ^43^ ^]^ Inflammation is recognized as an endogenous factor leading to senescence, while senescent cells tend to secrete “senescence‐associated secretory phenotypes” (SASPs) in response to inflammatory environments^[^ ^44^ ^]^; these cells can be considered sources of pro‐inflammatory factors and ROS, thus inducing senescence of healthy cells around in a positive feedback manner, leading to persistent senescence of tissues and inflammation dissemination. Tissue‐resident fibroblasts are mesenchymal cells and show strong plasticity in tissue regeneration. When tracheal substitutes are implanted in vivo, the peripheral inflammatory response is uncontrolled. There is evidence suggesting that several stresses related to the senescence process of fibroblasts, such as excessive inflammatory damage and oxidative stress, are inducers for their differentiation into myofibroblasts.^[^ ^45^ ^]^ Myofibroblasts are spindle‐shaped contractile cells that can secrete extracellular matrix components and a large number of pro‐inflammatory mediators.^[^ ^46^ ^]^ Thickening of the inner wall of the trachea and even narrowing have been proven to be associated with the continuous activation of myofibroblasts, leading to dysregulation of type I collagen remodeling.^[^ ^47^ ^]^ Therefore, in this study, quercetin, an anti‐inflammatory substance, inhibited the senescence of fibroblasts by regulating the inflammatory microenvironment, thereby terminating myofibroblast activity and promoting tissue repair to prevent the formation of pathological fibrous scars in the tracheal lumen. Moreover, in the inflammatory environment, the cartilage further accelerates disintegration, and the transmural vascular network cannot be established and is completely unable to meet the basic metabolic needs of the tissues in the early stage after transplantation, which will cause necrosis, ultimately resulting in a vicious cycle. Therefore, quercetin in the connective tissue site, which is the first area aggressively attacked by immune cells, was introduced to regulate regenerative homeostasis.

As the first line of host defense after tracheal substitute implantation, macrophages are heterogeneous and plastic^[^ ^48^ ^]^, and this work demonstrated that quercetin was capable of recruiting and reprogramming macrophages. Notably, macrophage polarization is a complex cellular process related to cellular metabolism. Mitochondria are important organelles that play important roles in cellular energy production and the immune response. Altering the immunometabolic profile is a potential strategy to activate macrophage polarization.^[^ ^24^, ^25^, ^26^ ^]^ As a by‐product of mitochondrial oxidative phosphorylation, ROS are capable of regulating cellular bioactivity, and Fan et al. demonstrated that excessive ROS promoted M1‐phenotype macrophage polarization in tendon repair, amplified inflammatory responses, and exacerbated the vicious cycle of an imbalanced immune microenvironment.^[^ ^49^ ^]^ In addition, excessive accumulation of ROS is considered a distinctive feature of senescent stem cells, which induces excessive oxidative stress, deteriorates the host regenerative environment, and ultimately becomes a key obstacle to the repair of defects in senescent tissues.^[^ ^50^ ^]^ However, there is still a lack of evidence demonstrating the specific effects of oxidative stress‐mediated mitochondrial damage and immune microenvironment imbalance on trachea repair and regeneration. Given quercetin's inherent chemically reductive structure (e.g., catechol moiety, 2,3‐double bond, and activated hydroxyl group on the heterocyclic ring), this work, for the first time, loaded quercetin to endow the scaffold with bioenergetic effects and explored its ability to reprogram macrophage by alleviating oxidative stress and maintaining the homeostasis of mitochondrial energy metabolism. In this way, macrophages provide anti‐senescence signals via paracrine mechanisms to ensure the phenotypes and biological functions of the cartilage, fibrous connective tissues, and vascular network, which may be key pathways for regenerating the complex tissues of the trachea.

Finally, we assembled the two types of rings designed for anti‐senescence into a tissue‐engineered trachea and embedded it in the paracervical region of New Zealand rabbits for pre‐vascularization before in situ transplantation. The results were consistent with those obtained from the in vitro study; the cartilage rings were neatly aligned and did not degrade, and the lumen of the trachea did not collapse, with good vascularization between the cartilage rings and epithelium coverage on the inner wall, resulting in good survival of the rabbits. In addition, senescence and inflammation‐related markers were not expressed in the experimental group, which confirmed that the entire trachea was in youth and free from inflammatory cells.

However, with regard to pre‐clinical experiments, before tracheal transplantation, the suitability of the animal model should always be carefully evaluated so as to obtain reliable and useful results from preclinical experiments. The selection of animal species as tracheal research models depends on multiple aspects, including operability, body size, cost, etc., but the most important is the anatomical, physiological, and genetic characteristics similar to those of humans.^[^ ^51^ ^]^ There are still differences in tracheal physiology between models of small animals (such as rats and rabbits), large animals (such as pigs and sheep), as well as humans. Overall, small animal models provide useful information for the feasibility of basic tracheal transplantation methods and the design and functional verification of tracheal grafts. However, there is still a considerable distance to go before more in‐depth efficacy analysis to promote clinical transformation. For example, the airway diameter of small animals is limited, and the operating space is insufficient. The characteristic of circumferential tracheal defects seems as higher technical complexity. Because operations like intubation need to be performed when the trachea is transected, the risk of death in small animal models is significantly increased, affecting the subsequent assessment of tracheal functions.^[^ ^52^ ^]^ In addition, small animals have a lower respiratory tidal volume, so the pressure of the airflow impacting the trachea wall is reduced, which puts forward higher requirements for the mechanical support capacity of the bioengineered trachea to be attempted to be used in large animals.^[^ ^53^ ^]^ For the reasons mentioned above, large animals such as pigs, whose anatomy, physiology, and immune systems are close to those of humans, will become the main animal models for our bioengineered tracheal research in the future. Large animals have a longer lifespan, which is convenient for evaluating the long‐term efficacy of bioengineered tracheal transplantation and identifying potential adverse events during treatment.^[^ ^54^ ^]^ However, if human clinical trials are conducted in the future, it is still necessary to compare and analyze the species similarities and differences between human and animal models. For example, unlike humans, the trachea of pigs is composed of complete cricoid cartilage with no posterior membrane wall. Moreover, the vascular anatomical structure is significantly different from that of humans, which may lead to adjustments regarding the surgical strategy for tracheal transplantation.^[^ ^55^ ^]^ In addition, postoperative management, such as preventing excessive stretching of the neck and postoperative complications, remains difficult to operate in large animal models.^[^ ^56^ ^]^ However, if clinical trials are conducted in the future, this is indispensable. Bronchial intervention may be required to address postoperative issues, such as granulation tissue hyperplasia, in order to improve patient survival rates. Finally, our goal is to achieve effective and ethical tracheal transplantation. Therefore, based on the Hebe‐ET in this study, it still needs to be optimized by referring to the complete clinical standards from cGMPs. The selected cells and tracheal scaffold materials, as well as the final composite substitute, all need to be prepared in accordance with verified and compliant standard operating procedures and should undergo regular quality control and batch‐to‐batch consistency tests.^[^ ^57^ ^]^ More data is required to prove the safety and effectiveness of the bioengineered tracheae. Only in this way can they obtain regulatory approval and be ultimately applied in clinical practice.

## 4 Conclusion

In summary, with regard to the key challenge of post‐transplanted dysfunction for bioengineered tracheae, this work emphasized senescence as the core mechanism and focused on two key factors: poor endogenous cellular contact and imbalance of the exogenous immune microenvironment. Then Hebe‐ET with a combination of physical and chemical strategies for anti‐senescence was innovatively constructed, and its long‐term functional maintenance was verified in the long‐segmental trachea defect model in rabbits, with highly native‐like morphological characteristics and multiple tissue regeneration (cartilage, transmural vascular network, and epithelium). Further research still needs to be carried out such as performing the repair of a larger tracheal defect (>6 cm) in a large animal model (such as a macaque or pig) and focusing on evaluating the long‐term survival outcome (>1 year) of the animal after tracheal reconstruction, which is an important research direction to push this tracheal replacement to clinical application.

## 5 Experimental Section

### Animals

New Zealand rabbits were purchased from Shanghai Jiagan Experimental Animal Company (Shanghai, China). SD rats and Balb/c nude (6‐week‐old) mice were purchased from Shanghai Slake Laboratory Animal Company. Animals’ routine care and study were carried out according to the guidelines of animal care and use (National Research Council and Tongji University). Procedures for the use of animals were approved by the Ethics Committee of Shanghai Pulmonary Hospital (Shanghai, China). We complied with all institutional and governmental regulations on animal ethics.

### Construction of the Hybrid Silk Fibroin Scaffold (HSFS)

For the preparation of SF non‐woven fiber, the raw silk (Jiaxing Silk Co. Ltd., China) was cut and combed, and the fiber mesh was formed by layered lapping. Subsequently, the collected SF mesh was fixed for needle punching (ZSZC01‐220, China), and the punching depth was 10 mm, which produced fiber entanglement inside the fiber mesh, turning the loose fiber mesh into enhanced non‐woven fiber fabric. The obtained non‐woven fibers were boiled in 0.02 m Na_2_CO_3_ solution for 2 h. After degumming, the fibers were washed with deionized water 5 times and then dried in an oven at 60 °C for 6 h. The dried non‐woven SF fibers were trimmed into cylinders with diameters ranging from 0.5 to 1 cm for subsequent experiments.

For the preparation of SF film, silk was dissolved in 9.3m lithium bromide solution (Macklin, China) after degumming treatment as described above to obtain SF solution, which was dialyzed with distilled water for 48 h and freeze‐dried to obtain porous SF cylinders. The cylinders were dissolved in distilled water to prepare homogeneous solutions with concentrations of 1, 3, and 5 wt%, and the solutions were added to the 48‐well plates in which the abovementioned non‐woven fibers were placed in advance. The plates were frozen at −20 °C overnight and subsequently freeze‐dried at −40 °C for 24 h. Finally, the freeze‐dried hybrid SF scaffolds were cross‐linked using anhydrous ethanol (Macklin, China) to obtain the final HSFS. In order to obtain the HSFS@Q, the mixed solution of silk protein and 5.3mg mL^−1^ quercetin (Macklin, China) was cast into the SF non‐woven fibers before freeze‐drying.

### Scanning Electron Microscopy (SEM)

Microstructure (top and side views) of HSFS was observed using SEM (JSM‐7001F, JEOL, Japan). Before imaging, a gold layer of 4 nm was applied to the sample surface using an auto‐sputter fine coater (JFC 1600, JEOL, Japan).

### Swelling and Volume Change Ratio Test

For the former, the dry weight of the scaffold (W_d_) was weighed at room temperature and then immersed in PBS. After removing the water from the surface of the scaffold, the wet weight (W_W_) was weighed. The formula for calculating the swelling ratio was: Swelling ratio (%) = (Ww – W_d_)/W_d_ ×100%. For the latter, the initial volume of the scaffold (V_d_) was measured (according to the cylinder diameter and height). After immersion in PBS, the scaffolds were removed, and the volume (Vw) was measured again. The formula for calculating the volume change ratio: Volume change ratio (%) = (Vw – V_d_)/V_d_ ×100%.

### Mechanical Performance

Cylindrical samples (10 mm in diameter and 5 mm in height) were prepared. The tensile properties were tested using an EZ‐LX universal testing machine (Shimadzu, Japan) with a clamping distance of 10 mm and a stretching speed of 10 mm min^−1^. Three samples were measured in each group. The microcomputer‐controlled electronic universal testing machine (WDW‐1, China) was used to test the compressive properties of the scaffold, the compressive speed was set at 10 mm min^−1^, and the deformation was controlled in the range of 0%–60%, and three samples were measured in each group.

### Hydrophilic Property

Hydrophilicity was evaluated by measuring the contact angle of deionized water droplets on the surface of the scaffold. The contact angles were measured by a contact angle meter (JC2000 DM, Shanghai).

### Water Vapor Transmission Rate (WVTR) Calculation

WVTR was used to evaluate the permeability of HSFS. Briefly, samples (surface area: S) divided the sealed space into upper and lower chambers. The upper chamber was vented with water vapor, which passed through the material and into the dryer in the lower chamber (initial mass: W_i_) at intervals of time (T), and the overall weight was changed (W_f_). The formula for calculating was: WVTR (g m^−2^· d^−1^) = 24×(W_i_ −W_f_)/S×T.

### In vitro Hemolysis Test

Hemolysis test was carried out to evaluate the hemocompatibility of HSFS. Fresh anti‐coagulated whole blood was washed three times and centrifuged to obtain precipitated red blood cells (RBC), and then mixed with PBS to obtain RBC suspension. Subsequently, HSFS was soaked in PBS for 24 h to obtain the leaching solution. 200 µL RBC suspension and 800 µL leaching solution were mixed and incubated at 37 °C for 1 h. The RBC suspension mixed with deionized water and PBS was used as negative and positive controls, respectively. Finally, the samples were centrifuged at 5000 rpm min^−1^ for 5 min (TG16, Shanghai), and the absorbance of the supernatant was measured at 540 nm using a microplate reader (Multiskan FC, USA), and the hemolysis rate (H) of the samples was calculated based on the absorbance value as: H (%) = A_s –_ A_ns_ / A_pc –_ A_ns_×100% (A_s_ represents the absorbance of the experimental samples, and A_pc_ and A_nc_ were the absorbance of the positive control group and the negative control group, respectively).

### Blood Clotting Test

The scaffolds were first placed in a 6‐well plate and incubated for 10 min at 37 °C. Then, 100 µL of rabbit whole blood was first dropped on the samples, followed by the addition of 20 µL CaCl_2_ solution (0.2 mol mL^−1^) immediately. 6‐well plates were then incubated at 37 °C for 20 min. Deionized water was added and incubated at 37 °C for another 5 min. Finally, the absorbance of the sample solution at 540 nm was measured, and the BCI was calculated as: BCI (%) = I_s_ / I_w_ ×100%. I_s_ represented the absorbance value of the experimental samples, and I_w_ represented that of the blank group.

### DPPH Clearance Assay

DPPH was dissolved in ethanol with a concentration of 100 µm. The scaffold leaching solution was obtained as described above. The two kinds of solution were mixed in equal volumes and incubated for 1 h free from light. The absorbance of the mixture at the wavelength of 517 nm was measured by the UV–visible spectrophotometer (UV1800, Japan). The formula for DPPH clearance was calculated as follows: DPPH clearance (%) = A_b_ ‐A_s_ /A_b_ ×100%. A_b_ is the absorbance of the blank sample, A_s_ is the absorbance of the experimental mixed solution.

### Chondrocyte Culture and In Vitro Biocompatibility of HSFS

The chondrocytes used in this study were derived from rabbit ears. Rabbits were anesthetized, and 4×6 cm^2^‐sized ears were harvested. After the tissue was sterilized with 75% ethanol, the skin and fascia attached to the rabbit ear were removed. Cartilage was cut into 1×1 mm^2^‐sized pieces and digested with 0.25% trypsin (Gibco, USA) for 20 min at 37 °C. After neutralizing and discarding the trypsin, cartilage fragments were submerged in 0.15% type II collagenase (Gibco, USA) for further digestion at 37 °C overnight. The cell suspension was passed through the 70 µm cell strainer (Falcon, USA) to remove tissue residues, and primary chondrocytes were collected by centrifugation. Primary chondrocytes (P0) were inoculated at a density of 1.2×10^6^ in 10 cm dishes and passaged to the second generation (P2) for subsequent studies. Chondrocytes were cultured in Dulbecco's Modified Eagle's Medium (DMEM, high glucose) (Pricella, China) supplemented with 100 U mL^−1^ penicillin–streptomycin solution (Biosharp, China) and 10% fetal bovine serum (FBS) (Corning, USA) in the cell incubator (37 °C and 5% CO_2_ concentration).

Chondrocytes were cultured in the scaffold leaching solution for 7 days, and then stained using a live/dead cell staining kit (Invitrogen, USA) and observed under the fluorescence microscope (Olympus IX73, Japan). The proliferative activity of the cells in the leaching solution was detected using a CCK‐8 kit (Dojindo, Japan) according to the manufacturer's instructions, and the absorbance value of the supernatant at 450 nm was measured.

### Regeneration of Cartilage Tissue In Vitro and In Vivo

HSFS were sterilized before cell inoculation: scaffolds were soaked in 75% ethanol, followed by PBS washing 2–3 times, and then irradiated under UV light. Subsequently, chondrocytes were inoculated into the scaffolds at a cell density of 3×10^7^ and incubated at 37 °C for 4 h, so that chondrocytes fully infiltrated and adhered to the scaffolds. Afterwards, the scaffolds were incubated with pro‐chondrogenic medium, which contained Insulin–Transferrin–Selenium (Gibco, USA) and pro‐chondrogenic growth factors, for 2 and 4 weeks in vitro. The culture medium was changed every other day to realize cartilage regeneration in vitro. After 4 weeks of in‐vitro culture, part of the cartilage tissues was implanted subcutaneously into the back of nude mice, and incubated for another 4 weeks to evaluate cartilage regeneration in vivo.

### Biochemical Analysis of Cartilage Tissue

Tissues were lyophilized in a vacuum, and cartilage biochemical assessment was performed according to the kit instructions. The DNA content of the tissues was determined using the TIANamp Genomic DNA Kit (Tiangen Biotech, Beijing). The samples were digested with papain at 65 °C for 12 h, and the GAG content was quantified using the Sulfated Glycosaminoglycan Assay Kit (Biocolor, UK). Finally, the HYP content of the tissues was determined by the alkaline hydrolysis method (Jiancheng, China).

### Induction of Macrophage Polarization In Vitro

Macrophages (RAW264.7 lineage, purchased from Pricella) were inoculated in 6‐well plates at a density of 2×10^5^ and cultured in complete medium after FBS inactivation (heated in a water bath at 56 °C for 30 min). After cells adhered to the wall, the culture conditions were changed with 1 mg mL^−1^ LPS (Sigma–Aldrich, USA), 1mg mL^−1^ IL4 & IL13 (PeproTech, USA), HSFS‐Q leaching solution (Macklin, China), and the mixture of LPS & leaching solution. After 5 days of continuous induction, macrophages were collected for subsequent assay.

### Flow Cytometry Analysis of Macrophage

To analyze macrophage polarization, cells were digested and collected (1×10^5^ per group), resuspended with buffer (PBS containing 2% FBS), and collected in 1.5 mL EP tubes. 200µL Fc‐Block was first added and incubated at 4 °C (for 10 min, and then buffer was added to terminate the reaction. Subsequently, macrophage‐specific antibodies were added and incubated at 4 °C for 20 min. All the antibodies were purchased from BioLegend (USA). Finally, cells were washed 2 times with buffer and resuspended. The samples were tested using a FACSVerse flow cytometer (BD Biosciences, Franklin Lakes, USA). Data were analyzed using FlowJo 10.6.2.

### ROS Level Assay

Following the manufacturer's instructions, DCFH‐DA (Beyotime, China) and MitoSOX Red (Invitrogen, USA) fluorescent probes were used to detect intracellular and mitochondrial ROS production levels in macrophages. Briefly, the cell culture medium was removed, and an appropriate volume (1–2 mL) of diluted DCFH‐DA and MitoSOX working solution was added to each well to incubate cells for 20 min at 37 °C. Afterward, cells were washed three times with PBS to remove residual fluorescent probes that had not entered the cells. Finally, cells were observed by light microscope and fluorescence microscope (Olympus IX73, Japan) alternately.

### Mitochondrial Membrane Potential Assay

Mitochondrial membrane potential assay kit with JC‐1 (Beyotime, China) was used according to the instructions. Briefly, macrophages were rinsed twice with PBS and then incubated with JC‐1 working solution for 20 min at 37 °C. Subsequently, the cells were washed twice with JC‐1 dilution buffer after ice bathing, and the images were captured using the fluorescence microscope immediately after the cell culture medium was added. The red/green fluorescence intensity was quantified using ImageJ software.

### Cellular ATP Production Level

ATP assay kit (Beyotime, China) was used to determine the ATP production level of macrophages. Add 200 µL lysis solution into each well to disintegrate macrophages at 4 °C, centrifuge at 12000×g for 4 min, collect supernatant (20 µL), and add ATP detection working solution (100 µL) into a 96‐well plate (black). The fluorescence intensity was read by the microplate reader. ATP concentration was calculated according to the standard curve.

### Mitochondrial Microstructure Morphology

After incubation, macrophages were obtained with cell scrapers. Cell microspheres were collected at the bottom of 1.5 mL EP tubes after centrifugation and fixed in 2.5% glutaraldehyde pre‐cooled at 4 °C overnight. Subsequently, the samples were rinsed with 1% osmium tetroxide and fixed for 1 h. After gradient dehydration in ethanol, the cell microspheres were embedded in resin to prepare ultrathin sections. Subsequently, the sections were stained with uranyl acetate and lead citrate. Finally, transmission electron microscopy (TEM) was used to observe whether the cellular mitochondria underwent morphological changes, such as vacuolization, cristae collapse, and mitochondrial swelling/disintegration.

### Construction of the Co‐Incubation Transwell Model In Vitro

To determine the potential paracrine impacts of macrophages on the regeneration of multiple tissues (cartilage, blood vessels, fibrous connective tissue) after polarization. Expanded macrophages were inoculated into the upper chamber of Transwell (Corning, USA) at a density of 5×10^4^ and cultured under the conditions described above (complete medium, supplemented with LPS, IL4&IL13, quercetin, and LPS‐quercetin mixture). Primary chondrocytes, HUVEC (Fuheng, China), and NIH‐3T3 mouse embryonic fibroblasts (Pricella, China) were inoculated at a density of 5×10^4^ into the lower chamber of Transwell. Cells were co‐cultured for 1 week, and their functions were subsequently examined.

### Cellular Senescence‐Associated β‐Gal Staining

β‐gal staining was performed on the above‐mentioned three cell lineages in the lower chamber of Transwell to detect cell senescence using the Senescence β‐Galactosidase Staining Kit (Beyotime, China). Briefly, aspirate cell culture medium, wash cells once with PBS, and then fix cells for 15 min at room temperature. Afterward, cells were washed three times for 3 minutes each time. Subsequently, the staining working solution was added to incubate cells at 37 °C overnight, and then cells were observed under the light microscope.

### Cell Cycle Assay

Cell cycle assay kit (Elabscience, China) was used to analyze the cell cycle progression of trilineage cells as mentioned above. Briefly, approximately 5×10^5^ cells were collected and centrifuged at 300×g for 5 min, and the supernatant was discarded. Subsequently, the cells were washed and resuspended with PBS, and then pre‐cooled −20 °C anhydrous ethanol was added, and the plate was placed at −20 °C overnight. After centrifugation, the supernatant was discarded, and the cells were resuspended in PBS and left at room temperature for 15 min. 100 µL RNase A Reagent was added and fully suspended, and then the cells were incubated at 4 °C without light for 30 min. The OD value was recorded at 488 nm.

### DNA Damage Detection

DNA damage assay kit by γ‐H2AX immunofluorescence (Beyotime, China) was used to analyze the DNA damage condition of trilineage cells as mentioned above. Briefly, cells were fixed in 4% paraformaldehyde for 15 min, then washed and immersed in blocking solution for 20 min. Cells were subsequently incubated with γ‐H2AX primary antibody overnight, washed three times with PBS, and then incubated with secondary antibody for 1 h. Cell nuclei were visualized by DAPI. Afterwards, Cells were observed under the fluorescence microscope, and ImageJ software was used to quantify the fluorescence intensity of positive cells.

### Toluidine Blue Staining

The secretion and distribution of chondrogenic glycosaminoglycans were detected using toluidine blue stain solution (Solarbio, China). Cells were rinsed twice with PBS, stained for 5 min, gently pipetted with distilled water, and let stand for 15 min. Finally, the cells were washed twice with distilled water and observed under the light microscope.

### Enzyme‐Linked Immunosorbent Assay (ELISA)

Concentrations of regeneration‐related cytokines, including pro‐chondrogenic growth factors (IGF‐1, TGF‐β1, and FGF‐2) and pro‐angiogenic factors (TGF‐β3, PDGF‐AA, and VEGF‐A), were determined in the conditional medium of macrophages using ELISA assay kits (Elabscience, China). All assays were performed according to the manufacturer's instructions. Briefly, a standard working solution or sample was first added and incubated at 37 °C for 90 min. Afterward, biotinylated antibody working solution, HRP enzyme conjugate working solution, substrate solution, and termination solution were added and incubated in sequence, and the OD value was read at 450 nm immediately.

### Scratch Wound Healing Assay

The scratch wound healing assay was performed to explore the migration ability of HUVECs. Briefly, when cells labeled with Dil grew to 70%–80% fusion, they were treated with 50 nmol mL^−1^ mitomycin for 4 h and washed twice with PBS. Subsequently, a slit was scratched in the cell monolayer with a sterile plastic tip, and cells were washed twice with PBS and incubated for 24 h under different culture conditions as described above. Scratches were imaged under the fluorescence microscope at four time points of 0, 6, 12, and, 24 h, respectively. The scratch closure area was calculated using ImageJ software.

### Tube Formation Assay In Vitro

The tube formation assay was performed to test the ability of HUVECs to form blood vessels. Briefly, the Matrigel (Corning, USA) was first prepared overnight at 0 °C and added to the bottom of pre‐cooled 48‐well plates in each well and incubated for 30 min at 37 °C, 5% CO_2_. HUVECs (3×10^5^) were inoculated into the gel and incubated for 6 h. The formation of capillary‐like structures was observed under light microscopy. The total number of tube junctions and tube length were subsequently analyzed and quantified using ImageJ software.

### Crystal Violet Staining

The Transwell inserts were placed in well plates, wetted with serum‐free medium before cell inoculation, and incubated at 37 °C and 5% CO_2_ for 6 h. Fibroblasts were digested and suspended with serum‐free medium, and the cell concentration was adjusted to 2×10^5^  mL^−1^. Conditioned mediums of macrophages were added to the lower chamber of the plate to serve as a chemoattractant, and cell suspensions were added to the upper chamber and co‐incubated for 24 h after being left in the clean bench for 20 min. Subsequently, the inserts were removed, the residual liquid in the upper chamber was aspirated, and the inserts were fixed in well plates with methanol solution at room temperature for 20 min. After that, the inserts were placed into well plates with crystal violet staining solution, and stained for 20 min at room temperature. The stained cells were washed with PBS, and the insert was wiped with a cotton swab to remove the cells that had not passed through the membrane. The bottom side of the inserts was placed upward and dried at room temperature, and finally observed under the light microscope.

### Real‐Time Quantitative PCR

Total cellular RNA was extracted using TRIzol reagent (Invitrogen, USA), and a NanoDrop 2000 spectrophotometer (Thermo Fisher Scientific, USA) was used to assess the integrity and purity of the extracted RNA. Transcription reversal DNA (cDNA) was performed to form a complementary according to the instructions of the commercial reverse transcription kit (Takara Bio, Japan). qPCR was performed to assess the expression levels of COL2, ACAN, COL1, COLX, CD31, VEGF‐α, and HIF‐1α. The primers are listed in Table S1 (Supporting Information).

### Scaffold Subcutaneous Embedding Model

SF@Q‐S with a diameter of 6 mm and a thickness of 2 mm were embedded subcutaneously on the back of SD rats for 2 and 4 weeks, respectively. The residual scaffold material and the surrounding tissues were sampled for subsequent study. SF‐S were used as controls.

### Construction of the Sandwich Model

As mentioned above, chondrocytes were inoculated with ring‐shaped HSFS, cultured in vitro for 4W to form the cartilage ring, placed between two HSFS@Q ring‐shaped scaffolds to form a “sandwich” structure (SF@Q‐SW), which was embedded in the paratracheal muscle of rabbits for 2 and 4 weeks. The middle cartilage layer and the upper and lower fibrous connective tissue layers were evaluated for subsequent functional assessment, respectively. HSFS without quercetin was used to replace HSFS@Q to construct the sandwich model (SF‐SW) as controls.

### Repair of Long‐Segmental Tracheal Defects in Rabbits

New Zealand rabbits weighing 2 kg were selected for the repair of long tracheal defects. A cartilage ring and a fibrous connective tissue ring were constructed by inoculating chondrocytes and loading quercetin in the HSFS, respectively. Subsequently, the two rings were alternately stacked on a silicone tube to form the Hebe engineered trachea (Hebe‐ET), and the biomimetic trachea was wrapped with cervical muscles for pre‐vascularization. The natural trachea with the same length as Hebe‐ET was circumferentially resected to create the long segmental tracheal defect. After pre‐vascularization for 4 weeks, Hebe‐ET with vascular pedicles was separated from the surrounding tissues and transplanted to the tracheal defect site. End‐to‐end anastomosis was performed using 4‐0 absorbable suture (Jinhuan, China). To avoid infection, animals underwent intramuscular injection with penicillin sodium (40 000 U kg^−1^) every day for altogether 7 days, and the animals were executed 12 weeks after tracheal transplantation, and the engineered tracheae were sampled for subsequent analysis. Meanwhile, HSF‐ET was used as a control.

### Respiratory Function Monitoring

The respiratory rate of the animals was monitored once a week, which was recorded during a 10‐min observation period at three different time points of the day (7:00 a.m., 12:00 p.m., and 7:00 p.m.). Before each test, the animals were acclimatized for 15 min to ensure that they were in a calm, undisturbed state. The total number of respiratory breaths recorded at the three time points was averaged as the respiratory rate of the animal for that day.

### Histological and Immunofluorescent Staining Analysis

Tissues were fixed in 4% paraformaldehyde for 24 h, followed by gradient dehydration in ethanol, and were prepared as paraffin samples and set at a thickness of 8 µm for tissue sectioning. H&E, Safranine‐O, Alcian Blue, and Sirius Red staining were performed using the staining kits (Solarbio, China). Staining was performed according to the recommended procedure. The expression of CD86, CD206, F4/80, F‐actin, P16, P21, HMGB1, CK14, FOXJ1, α‐SMA, CD31, COL2, and MMP13 were detected by immunofluorescence staining under the standard IF protocol as follows: all samples were washed with PBS and subsequently permeabilized in 0.3% TritonX‐100 (Sigma–Aldrich, USA) for 5 min, then washed with PBS and blocked with 5% BSA (Sangon Biotech, China). Tissues were subsequently incubated with primary antibody overnight, washed three times with PBS, and then incubated with secondary antibody for 1 h. Cell nuclei were visualized by DAPI. Afterwards, tissue sections were observed under the fluorescence microscope, and ImageJ software was used to quantify the fluorescence intensity or the proportion of positive cells.

### Statistical Analysis

All the data were presented as mean ± standard deviation (SD) values. Data were analyzed using GraphPad Prism software (10.3). One‐way analysis of variance (ANOVA) was used to determine the level of significance between three or more groups. Differences between the two groups were analyzed using an independent samples t‐test. In all cases, *p* < 0.05 was considered statistically significant.

## Conflict of Interest

The authors declare no conflict of interest.

## Supporting information



Supporting Information

Supplemental Movie 1

Supplemental Movie 2

Supplemental Movie 3

## Data Availability

The data which supports the findings of this study is available from the corresponding authors upon reasonable request.
