# Supplementary material for: Engineered Anti‐Senescence Trachea With Post‐Transplanted Regenerative Homeostasis
Source: Adv Sci (Weinh). 2025 Jul 6;12(36):e07186. doi: 10.1002/advs.202507186 (PMC12462952; doi:10.1002/advs.202507186)
Supplement: Supplementary file 1 — Supporting Information [file ADVS-12-e07186-s002.docx]

Supporting Information

Engineered Anti-senescence Trachea with Post-transplanted Regenerative Homeostasis

*Ziyin Pan ^1,2,†^, Hai Tang ^1,2,†^, Lanlan Wang ^5,†^, Qingfeng Bai ^1,2^, Yi Chen ^1,2^, Runfeng Cao ^1,2^, Weikang Lin ^6^, Lei Wang ^1,2^, Yulong Hu ^1,2^, Guofang Zhao ^7^, Minglei Yang ^7, 8^, Weiyan Sun ^1,2,⁎^, Kun Zhang ^4,⁎^, Dawei Li ^3,⁎^, Chang Chen ^1,2,⁎^*


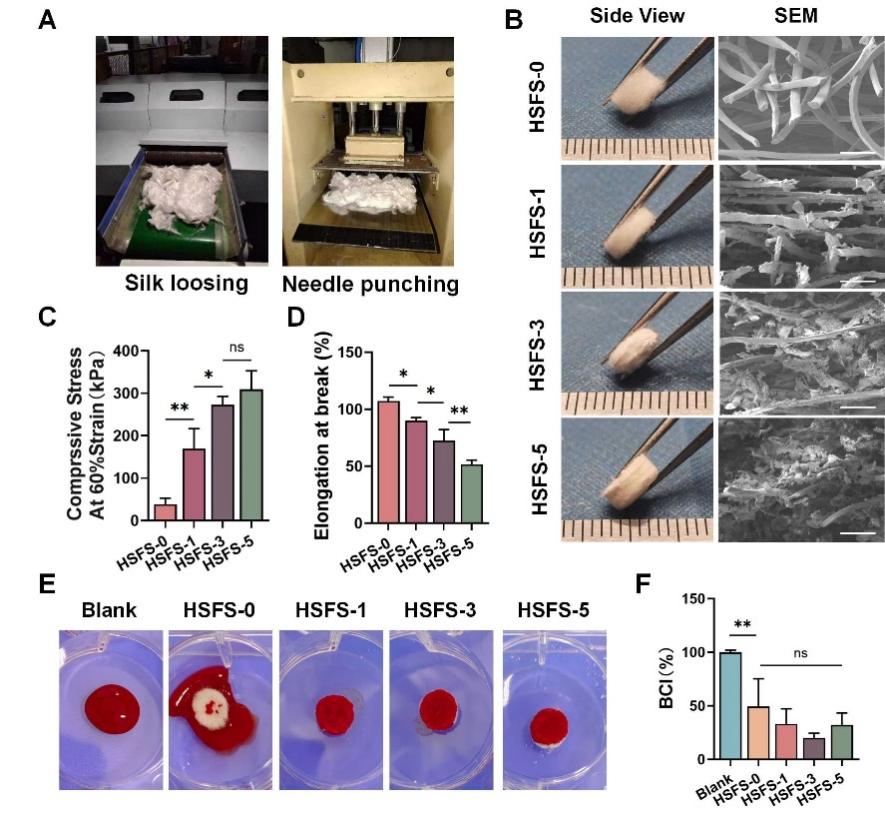


**Figure S1. Additional data related to the preparation and characterization of the “fiber-film” hybrid silk fibroin scaffolds (HSFS). A.** Scene of silk raw material undergoing the silk loosing and needle punching operations. **B.** Gross side view and SEM images of scaffolds with different preparation parameters (HSFS-0,1,3,5). Scale bar: 50μm. **C.** Compressive stress at 60% strain. n=3. **D.** Elongation ratio of the scaffolds at tensile rupture. n=3. **E.** Photograph record of in-vitro blood coagulation test and quantification of blood clotting index. n=3. N represent biological independent samples. The P values were determined using one-way ANOVA followed by Tukey’s multiple comparisons test. *P<0.05, **P<0.01.


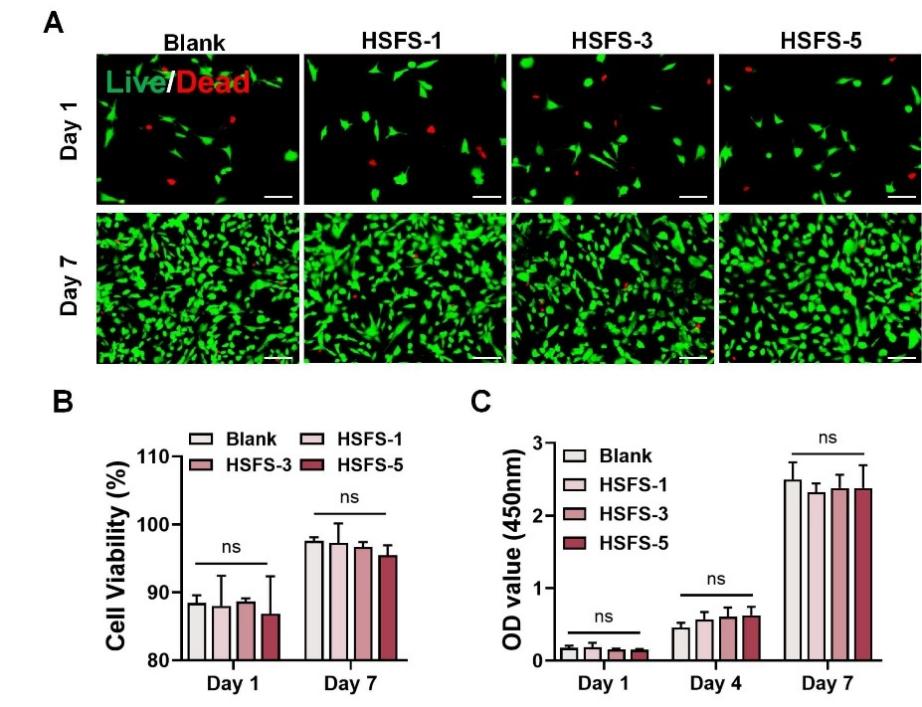


**Figure S2. Biocompatibility evaluation of the scaffold with rabbit primary chondrocytes. A.** Live/dead staining of the primary rabbit chondrocytes cultured with the leaching solutions of scaffolds (HSFS-0,1,3,5) , and the complete medium was used as control. Scale bar: 100μm. **B.** Quantitative analysis of living cells based on Figure A. n=3. **C.** Cell counting Kit-8 was used to detect the proliferation activity of primary rabbit chondrocytes cultured with the leaching solutions of scaffolds (HSFS-0,1,3,5). n=3. N represent biological independent samples. The P values were determined using two-way ANOVA followed by Tukey’s multiple comparisons test.


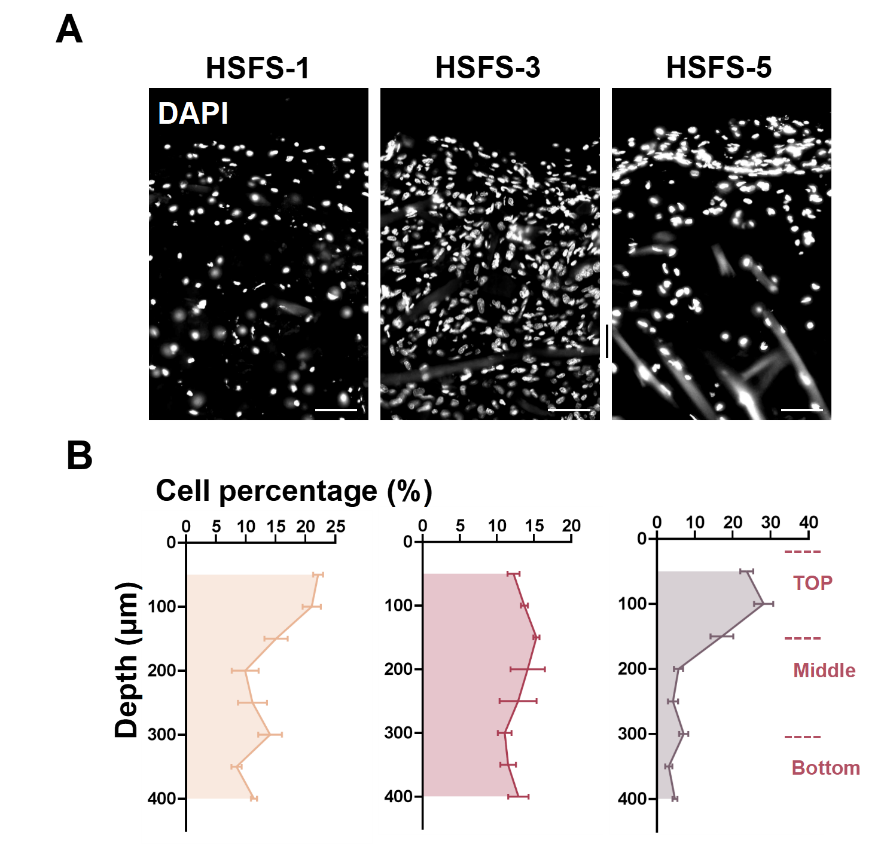


**Figure S3. Penetration conditions of chondrocytes after seeding on HSFS. A.** DAPI fluorescence staining of primary rabbit chondrocytes on scaffolds (HSFS-0,1,3,5). Scale bar: 50μm. **B.** The quantitative map of cell distribution proportion at different infiltration depths (50μm-400μm) based on figure A. n=3 at each depth. N represent biological independent samples. The P values were determined using one-way ANOVA followed by Tukey’s multiple comparisons test.


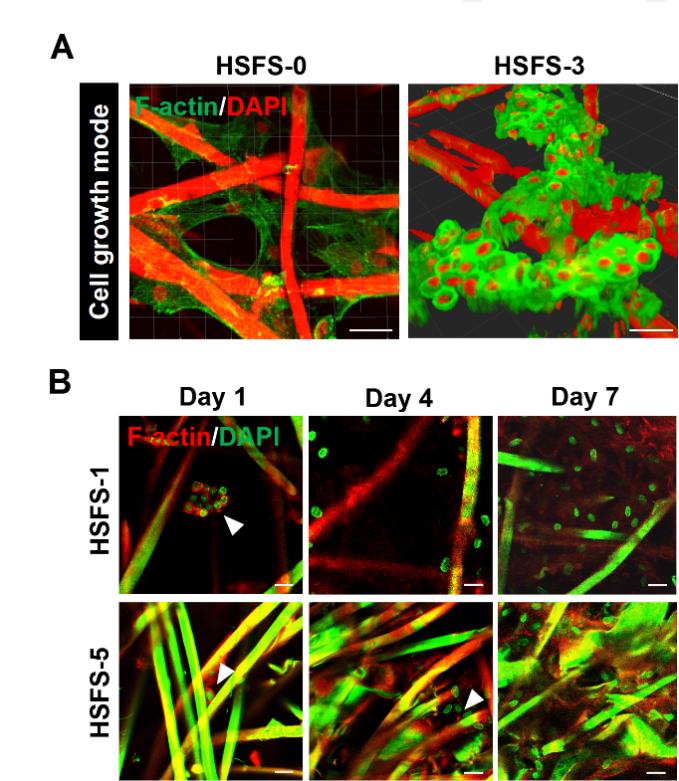


**Figure S4. Visualization of cartilage growth on HSFS. A.** Representative 3D reconstruction images of chondrocytes growing on non-woven fiber scaffold (HSFS-0) and HSFS-3. F-actin (red)/DAPI (green) fluorescence staining. Scale bar: 30μm. **B.** On the 2D plane, typical F-actin (red)/DAPI (green) fluorescence staining images of chondrocytes growing on HSFS-1, 5 for 1, 4, and 7 days respectively. Scale bar: 25μm.


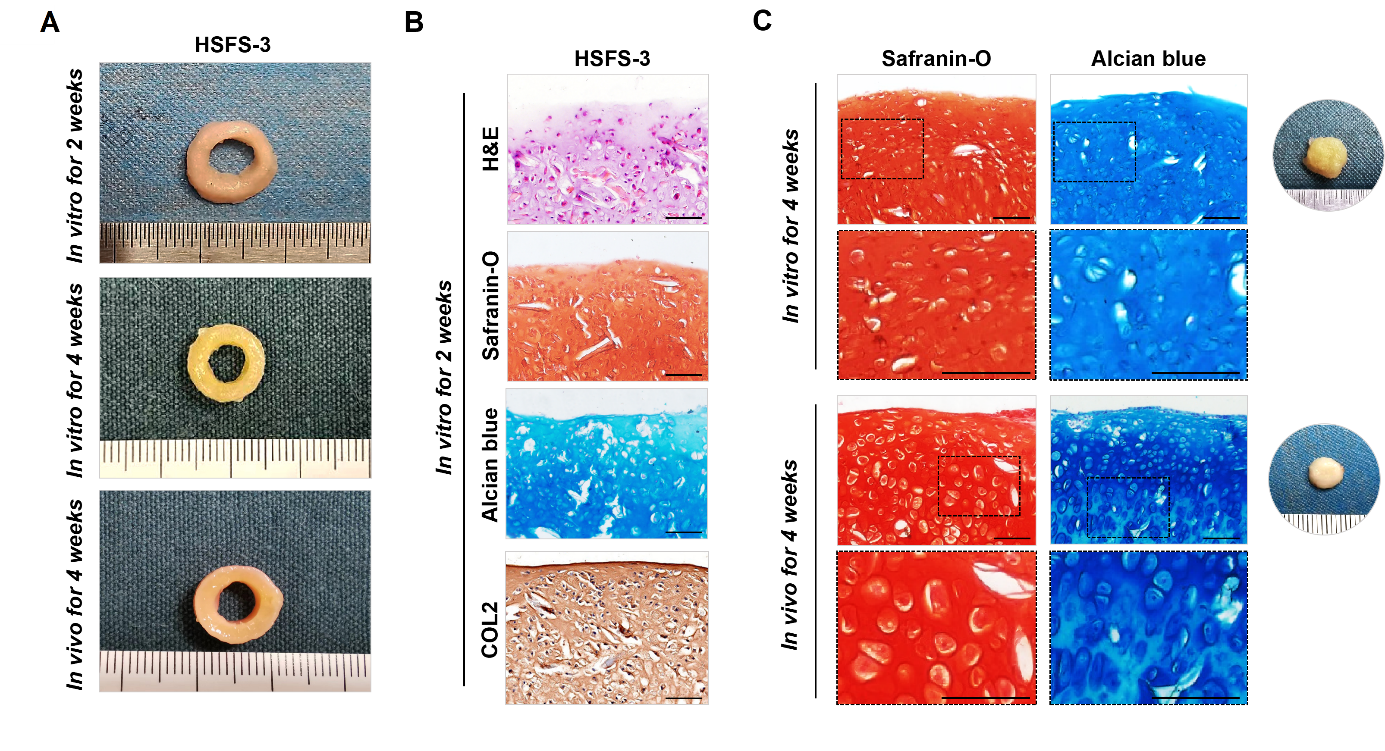


**Figure S5. Cartilage regeneration after seeding chondrocytes on HSFS-3 for 2W and 4W in vitro and embedded into Balb/c nude mice for continuous 4W culture in vivo. A.** Appearance of cartilage ring. **B.** H&E, safranin-O, alcian blue and COL2 IHC staining images of cartilage after 2W culture in vitro. **C.** The general appearance, safranin-O and alcian blue staining images of cartilage after 4W culture in vitro and in vivo. Scale bar: 100μm.


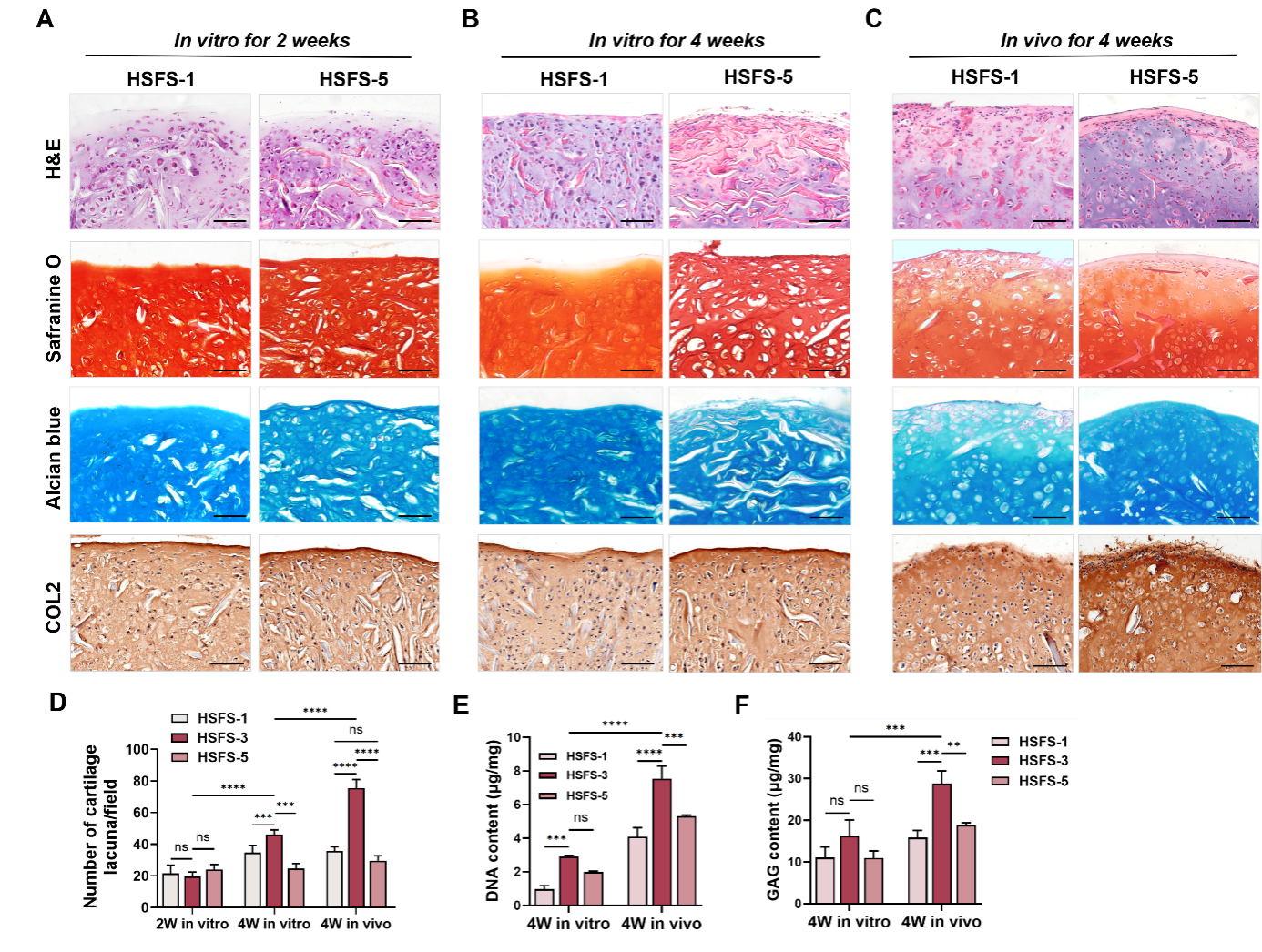


**Figure S6. Histological staining and biochemical quantification of cartilage after chondrocytes were seeded on HSFS and cultured for different time. (A-C)** H&E, safranin-O, alcian blue and COL2 IHC staining images of cartilage cultured in vitro for 2W **(A)**, 4W **(B)** and in vivo for 4W further culture **(C)**. Scale bar: 100μm. **D.** Cartilage lacuna count. n=5. **(E-F)** DNA content **(E)** and GAG content **(F)** of the cartilage. n=3. N represent biological independent samples. The P values were determined using two-way ANOVA followed by Tukey’s multiple comparisons test. *P<0.05, **P<0.01, ***P<0.001, ****P<0.0001.


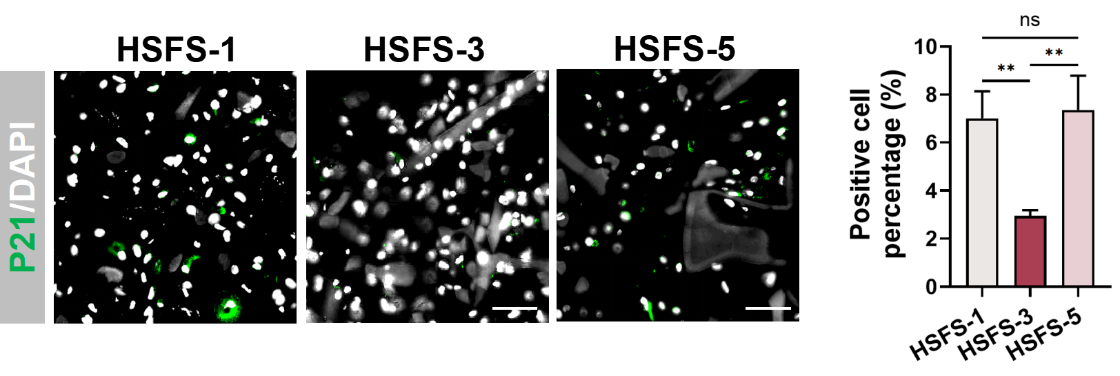


**Figure S7.** P21 (green)/DAPI (white) fluorescence staining of the cartilage cultured in vivo for continuous development, and positive cell proportion quantification. Scale bar: 50μm. n=3. N represent biological independent samples. The P values were determined using one-way ANOVA followed by Tukey’s multiple comparisons test. **P<0.01.


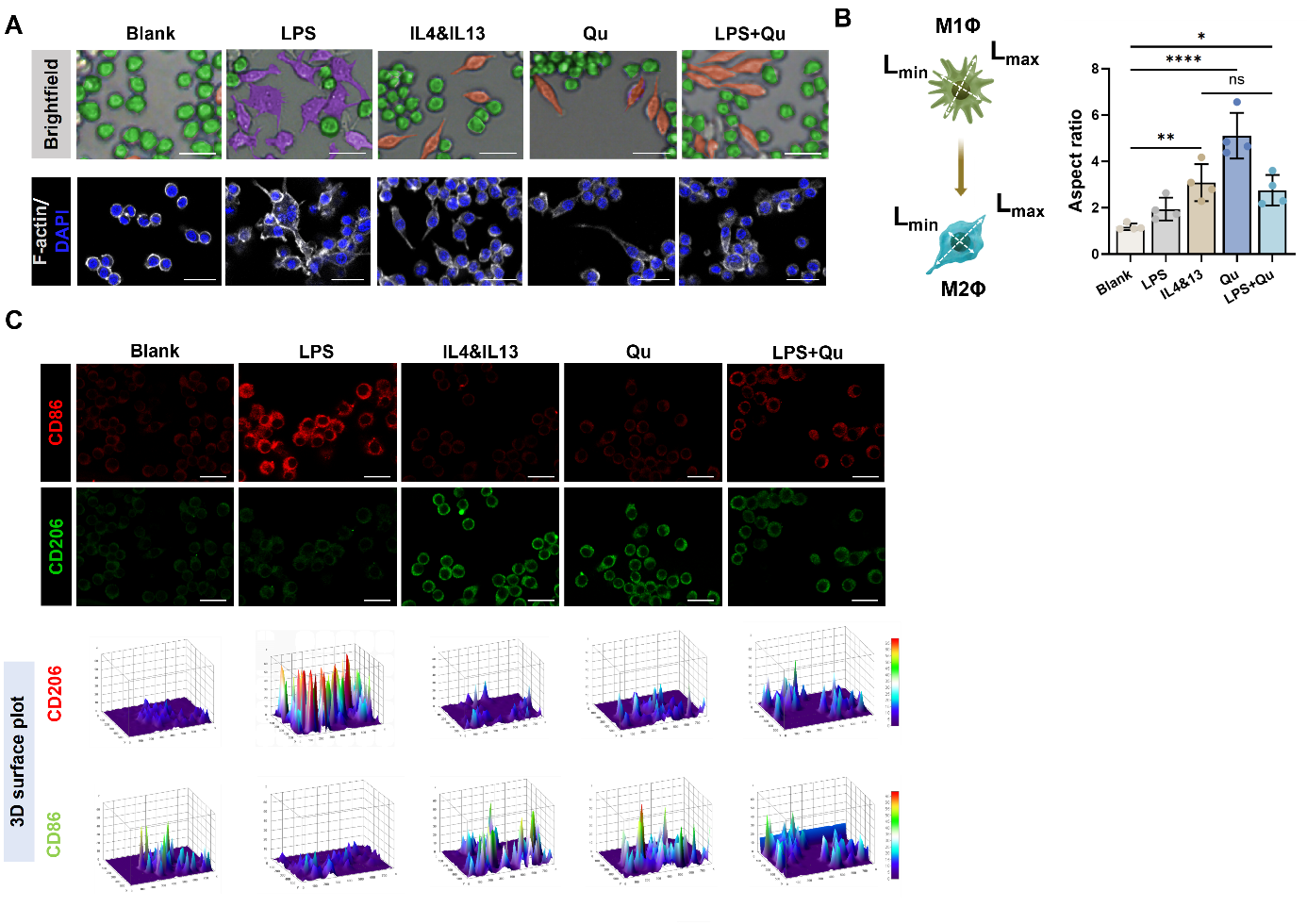


**Figure S8. Polarization induction of quercetin on macrophages. A.** Cell morphology observation of RAW 264.7 macrophages under light microscope and cytoskeleton fluorescence staining (white). Scale bar: 25μm. **B.** Morphological analysis of macrophages in Figure A. Aspect ratio refers to the ratio of long axis and short axis of cells. n=4. **C.** IF staining: CD86 (red), CD206 (green) and nucleus (DAPI, blue), and the 3D surface plot quantifying the fluorescence intensity of CD86 (red) and CD206 (green). Scale bar: 25μm. N represent biological independent samples. The P values were determined using one-way ANOVA followed by Tukey’s multiple comparisons test. *P<0.05, **P<0.01, ****P<0.0001.


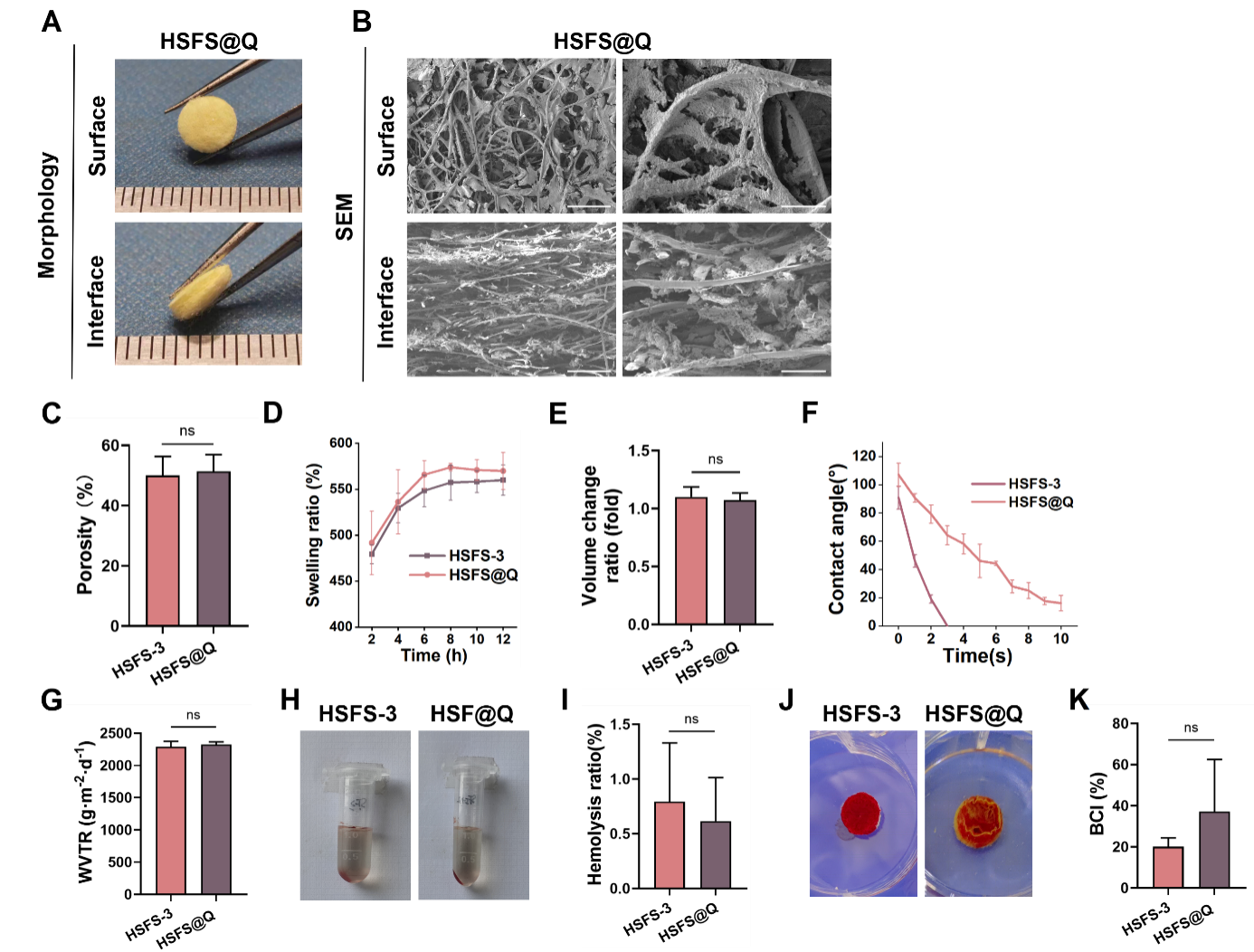


**Figure S9. Characterization of HSFS loaded with quercetin (HSFS@Q). A.** General morphology of the scaffold. **B.** SEM images and representative enlarged field. Scale bar: 250μm (low magnification view) and 50μm (high magnification view). **C.** Porosity calculation **D.** Swelling curves of the scaffold after immersion in PBS. **E.** The volume change of the scaffold in the wet environment. **F.** Contact angle change curves within 10 seconds. **G.** Quantification of WVT rate of scaffolds. **(H-I)** Photograph record and hemolysis ratio quantification of in vitro blood compatibility test. **(J-K)** Photograph record of in-vitro blood coagulation test and quantification of blood clotting index. n=3. N represent biological independent samples. The P values were determined using one-tailed unpaired t-test followed by Tukey’s multiple comparisons test.


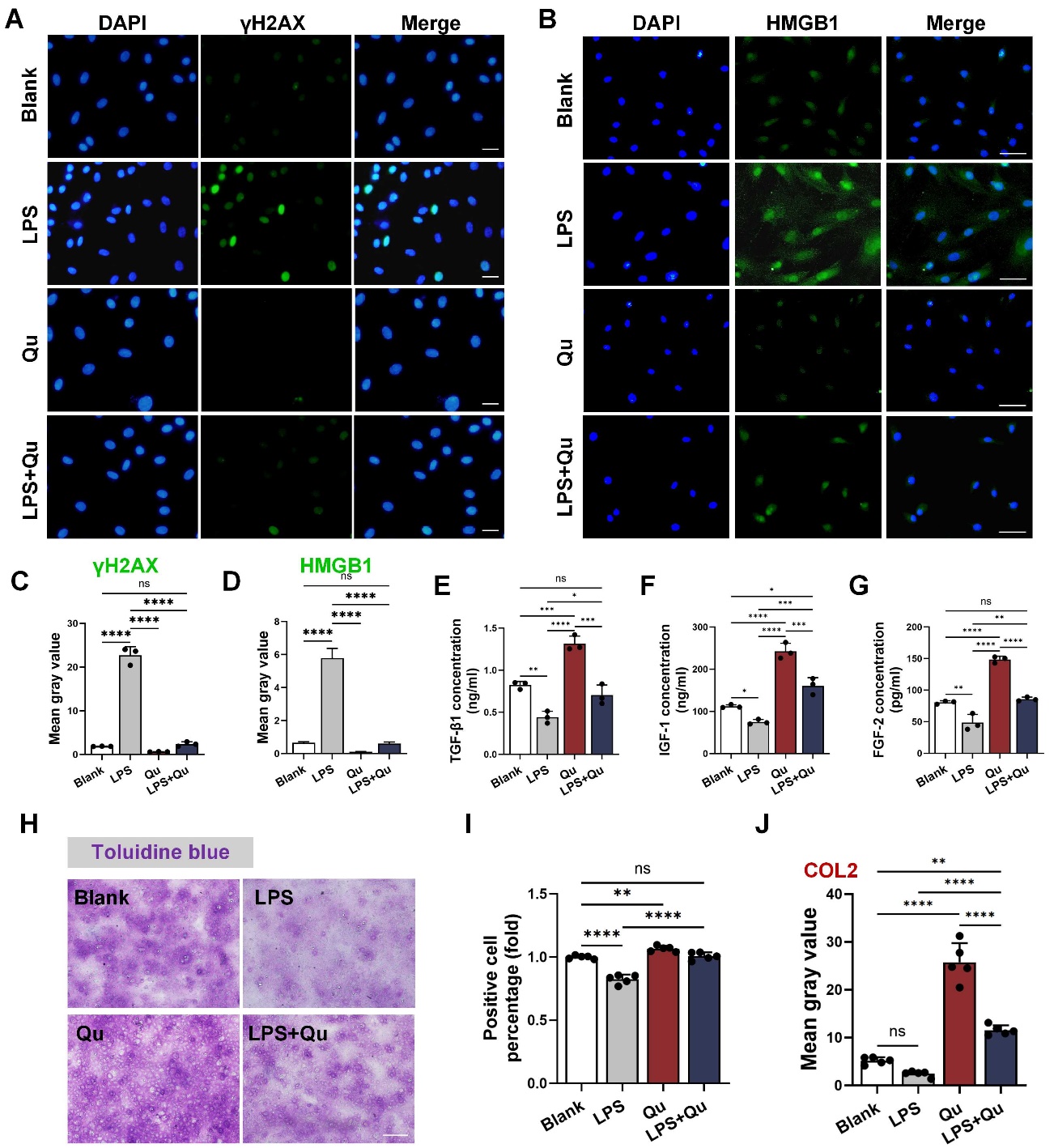


**Figure S10. In vitro validation of quercetin promoting chondrocyte development through the immune-senescence cascade. (A-D)** γH2AX IF staining **(A)**, HMGB1 IF staining (B), and quantification of fluorescence intensity, respectively **(C, D)**. Scale bar: 50μm. n=3. **(E-G)** The concentration of pro-chondrogenic cytokines: TGF-β1 **(E)**, FGF-2 **(F)** and IGF-1 **(G)** in macrophage conditioned medium measured by Elisa kit. n=3. **(H-I)** Toluidine blue staining and positive area ratio. Scale bar: 200μm. n=5. **J.** COL2 fluorescence intensity of chondrocytes under different culture conditions. n=5. N represent biological independent samples. The P values were determined using one-way ANOVA followed by Tukey’s multiple comparisons test. *P<0.05, **P<0.01, ***P<0.001, ****P<0.0001.


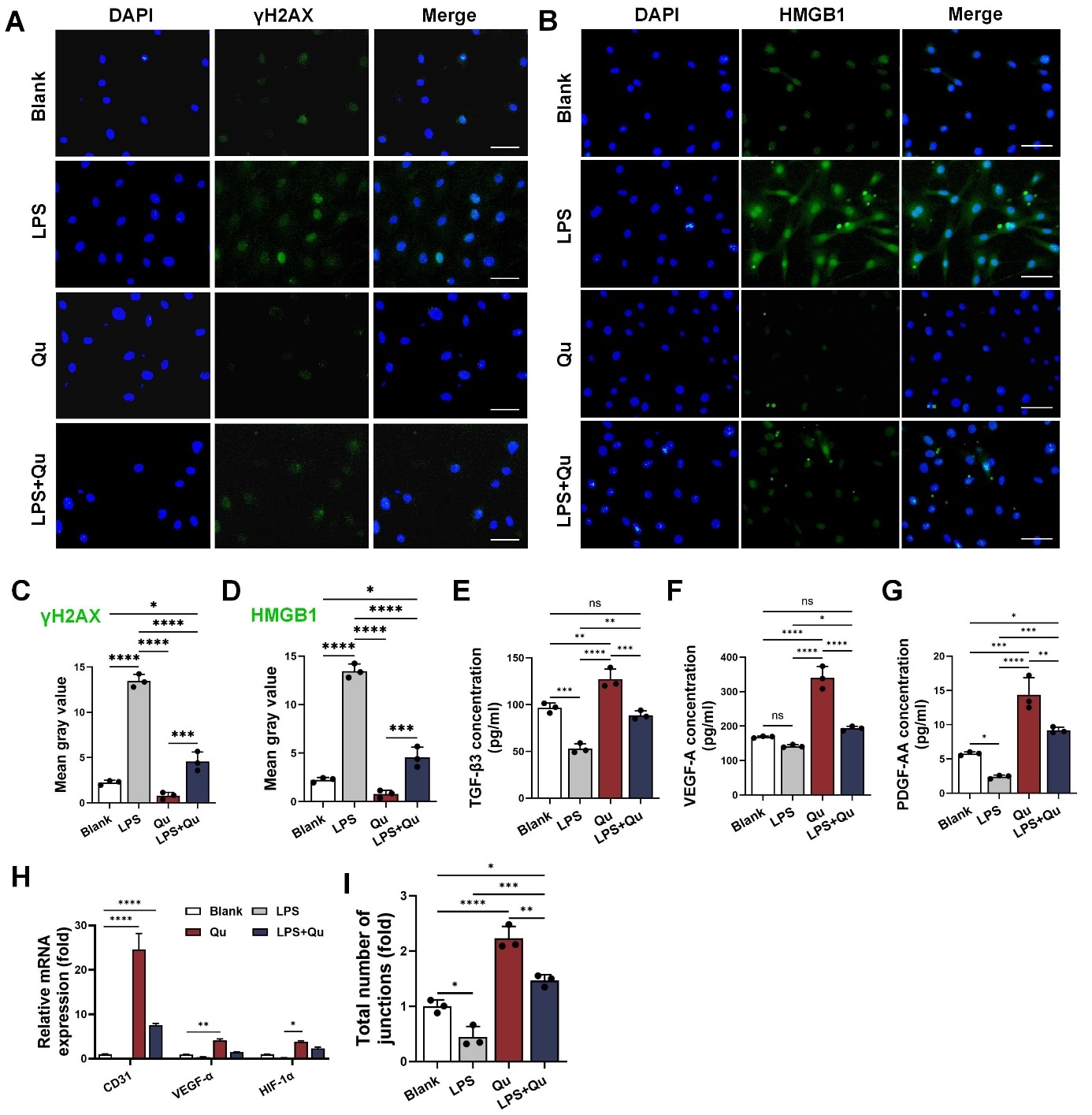


**Figure S11. In vitro validation of quercetin promoting endothelial cell development through the immune-senescence cascade.** **(A-D)** γH2AX IF staining **(A)**, HMGB1 IF staining **(B)**, and quantification of fluorescence intensity, respectively **(C, D)**. Scale bar: 50μm. n=3. **(E-F)** The concentration of pro-angiogenic cytokines: TGF-β3 **(E)**, VFGF-A **(F)** and PDGF-AA **(G)** in macrophage conditioned medium measured by Elisa kit. n=3. **H.** The expression of vascular related genes (CD31, VFGF-A and HIF-1α) measured by qPCR, n=3. **I.** Total number of vascular junctions in the tube forming experiment, n=3. N represent biological independent samples. The P values were determined using one-way or two-way ANOVA followed by Tukey’s multiple comparisons test. *P<0.05, **P<0.01, ***P<0.001, ****P<0.0001.


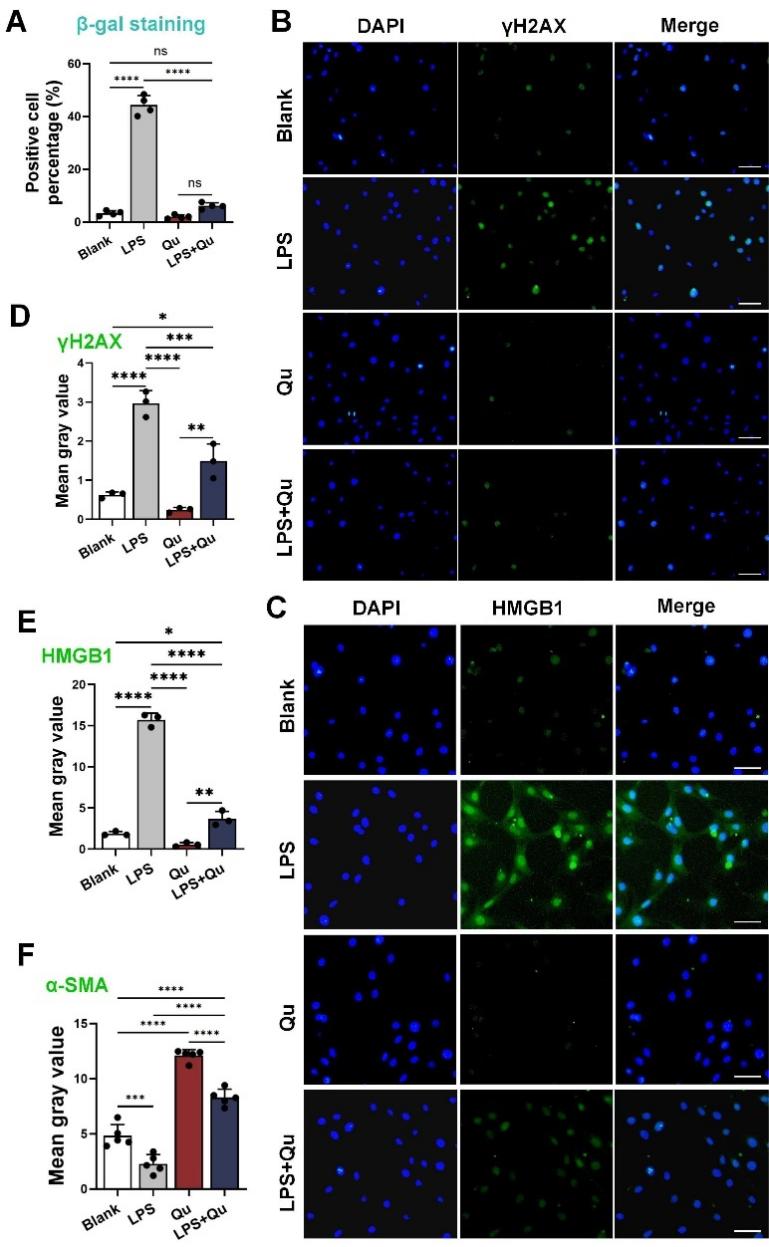


**Figure S12. In vitro validation of quercetin promoting fibroblast development through the immune-senescence cascade. A.** The proportion of β-gal positive cells in fibroblasts was quantified. n=4. **(B-E)** γH2AX IF staining **(B)**, HMGB1 IF staining **(C)**, and quantification of fluorescence intensity, respectively **(D, E)**. Scale bar: 50μm. n=3. **F.** α-SMA fluorescence intensity of fibroblasts under different culture conditions. n=5. N represent biological independent samples. The P values were determined using one-way or two-way ANOVA followed by Tukey’s multiple comparisons test. *P<0.05, **P<0.01, ***P<0.001, ****P<0.0001.


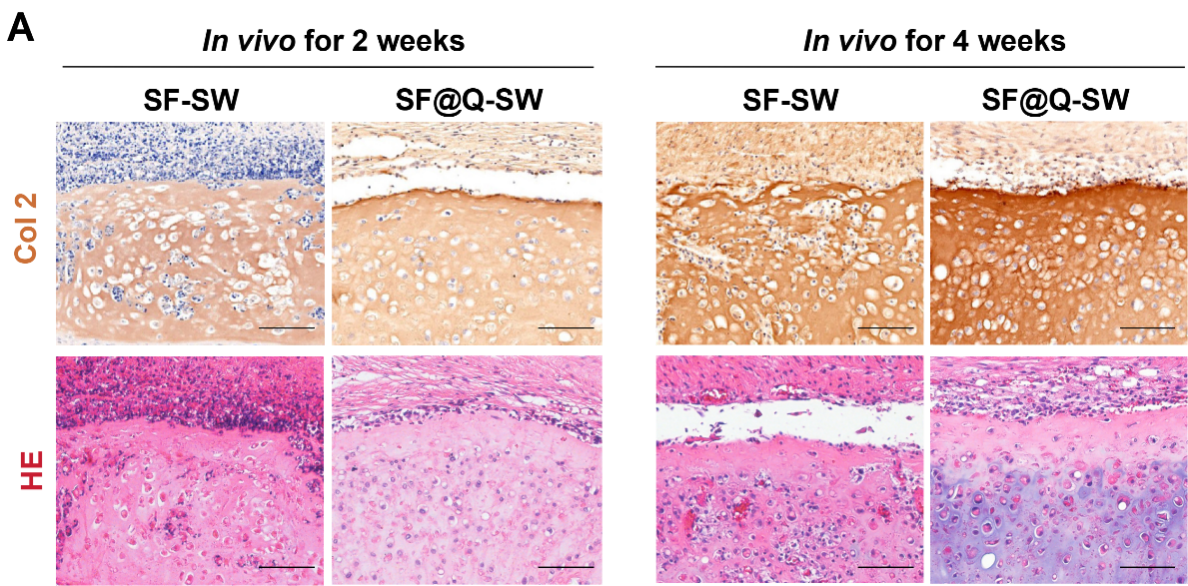


**Figure S13. A.** COL2 IHC and H&E staining of sandwich tissues (SF-SW and SF@Q-SW) after embedding (including the fibrous connective tissue above and the cartilage below). Scale bar: 100μm.


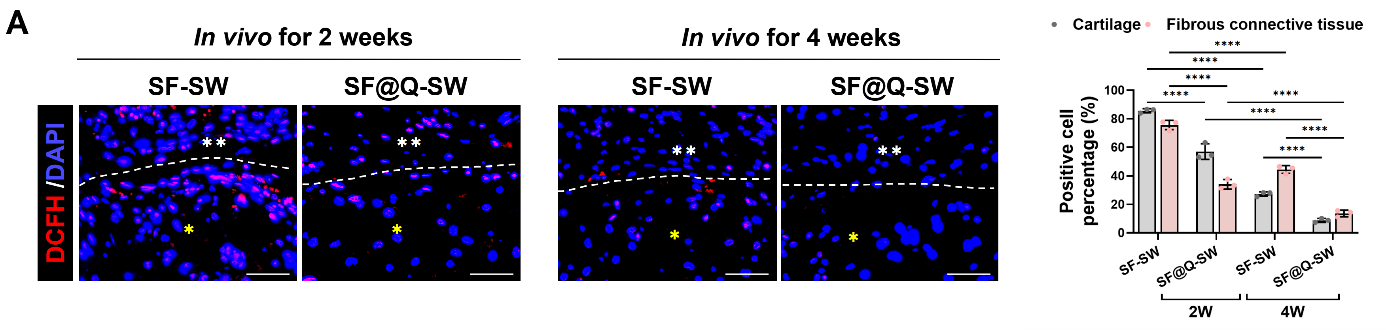


**Figure S14. A.** DCFH IF staining and positive cell percentage, white dotted line represents the boundary between cartilage and fibrous connective tissue. The double white asterisk represented connective tissue, and the single yellow asterisk represented cartilage. Scale bar: 50μm. The P values were determined using one-way or two-way ANOVA followed by Tukey’s multiple comparisons test. ****P<0.0001. n=3. N represent biological independent samples.


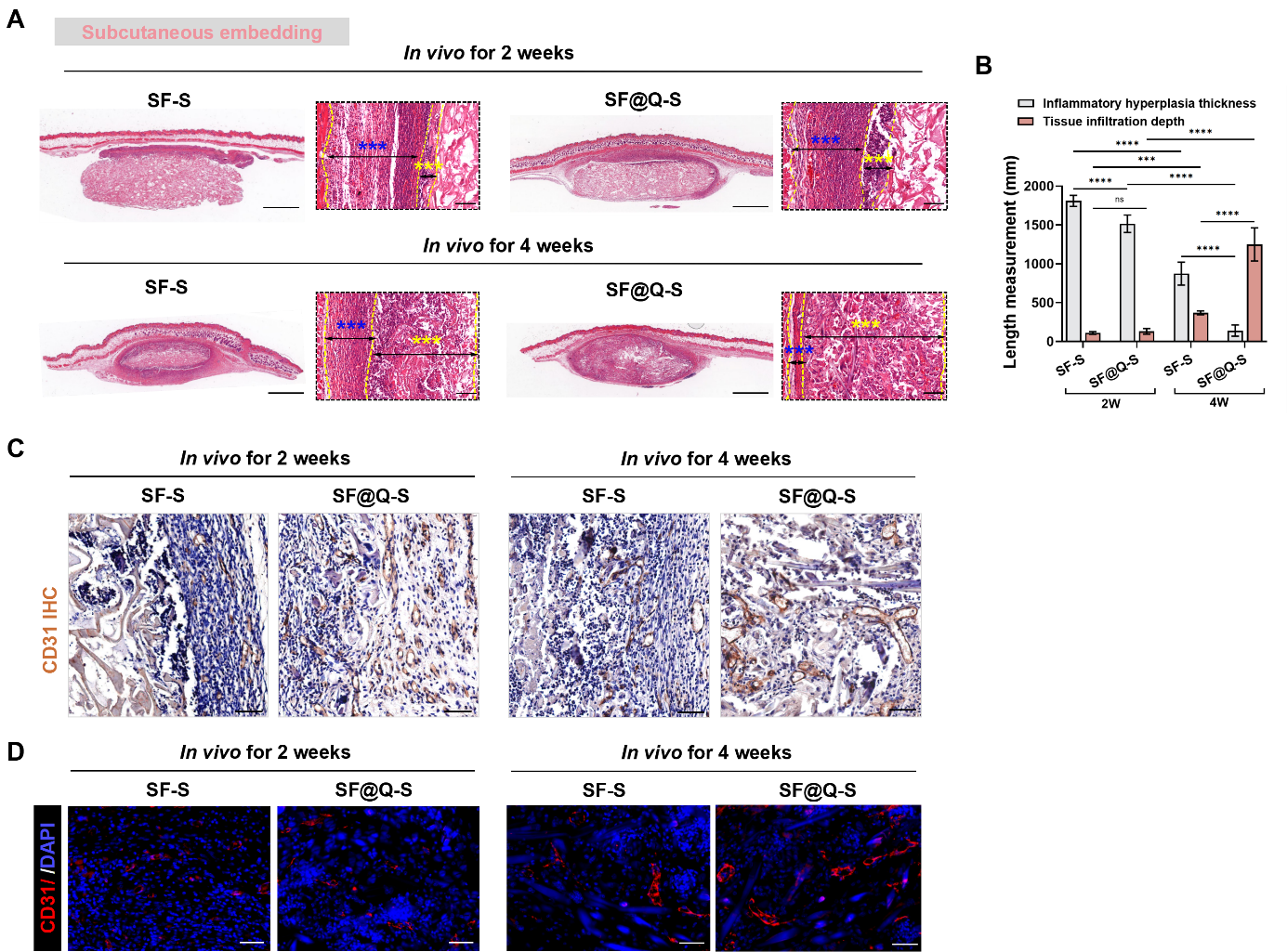


**Figure S15. Tissue infiltration and vascular regeneration in SF-S and SF@Q-S after subcutaneous scaffold embedding in rats for 2 and 4 weeks. A.** The H&E staining images of the scaffolds loaded with/without quercetin (SF-S and SF@Q-S) after subcutaneous implantation on the back in rats. Representative areas were locally magnified. The dotted yellow line represents the boundary of inflammatory hyperplasia layer and tissue infiltration layer. The blue and yellow asterisks as well as the double arrows represent the inflammatory hyperplasia layer, and the tissue infiltration layer, respectively. Scale bar: 1.5mm (low magnification view) and 100μm (high magnification view). **B.** Thickness quantification of the inflammatory hyperplasia layer and the tissue infiltration layer after subcutaneous scaffold embedding in rabbits. n=8. N represent biological independent samples. **C.** CD31 IHC staining. Scale bar: 50μm. **D.** CD31 IF staining. Scale bar: 100μm. The P values were determined using one-way or two-way ANOVA followed by Tukey’s multiple comparisons test. *P<0.05, **P<0.01, ***P<0.001, ****P<0.0001.


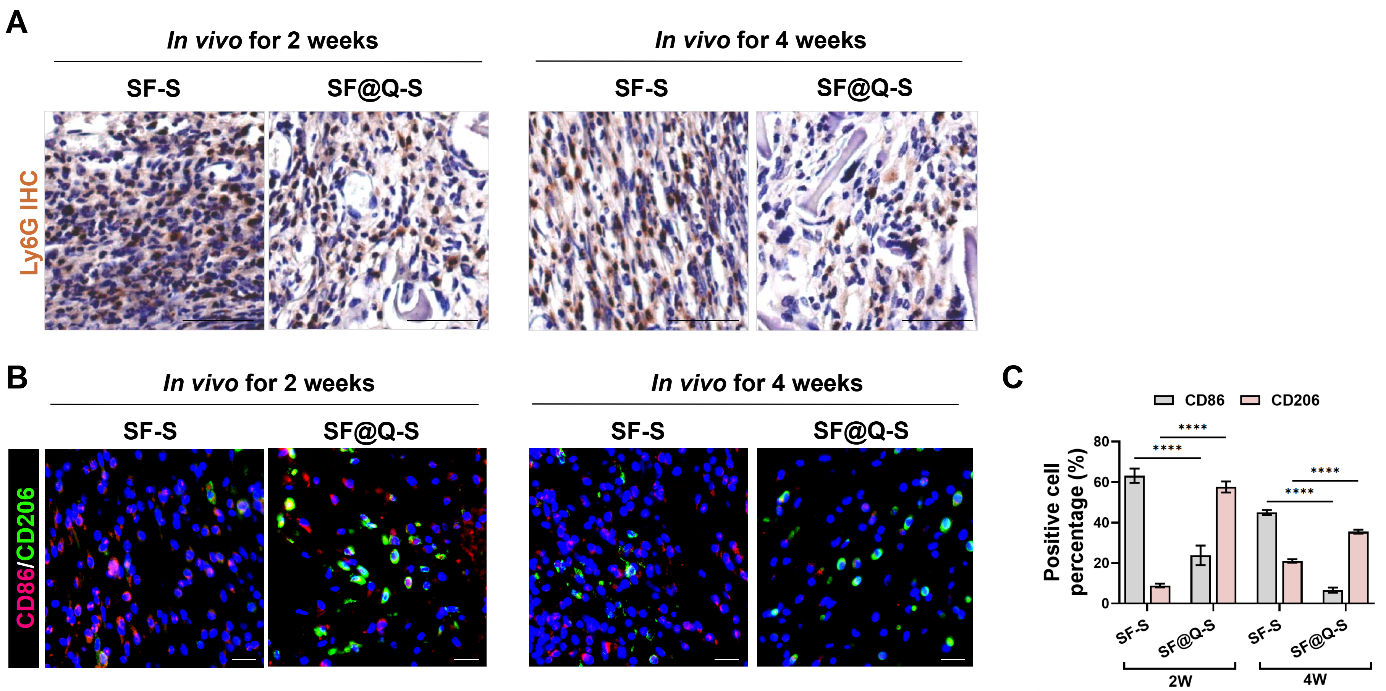


**Figure S16. Immune responses in SF-S and SF@Q-S after subcutaneous scaffold embedding in rats for 2 and 4 weeks. A.** IHC staining of Ly6G (neutrophil marker). **(B-C)** IF staining and positive cell percentage of macrophage related indicators CD86 (red) and CD206 (green). Scale bar: 20μm. n=3. N represent biological independent samples. The P values were determined using two-way ANOVA followed by Tukey’s multiple comparisons test. ****P<0.0001.


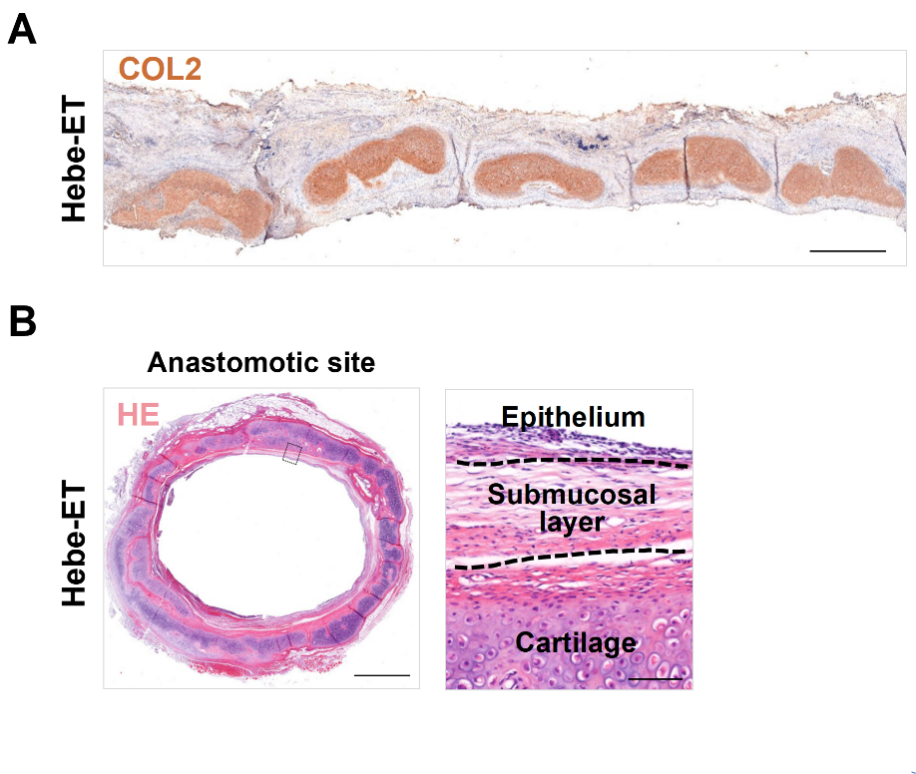


**Figure S17.** **Assessment of tissue regeneration after orthotopic transplantation of the Hebe engineered trachea (Hebe-ET). A.** COL2 IHC staining of representative longitudinal section of Hebe-ET. Scale bar: 1mm. **B.** H&E staining of cross section of Hebe-ET and representative magnified view. Scale bar: 1.5mm (low magnification view) and 80μm (high magnification view).


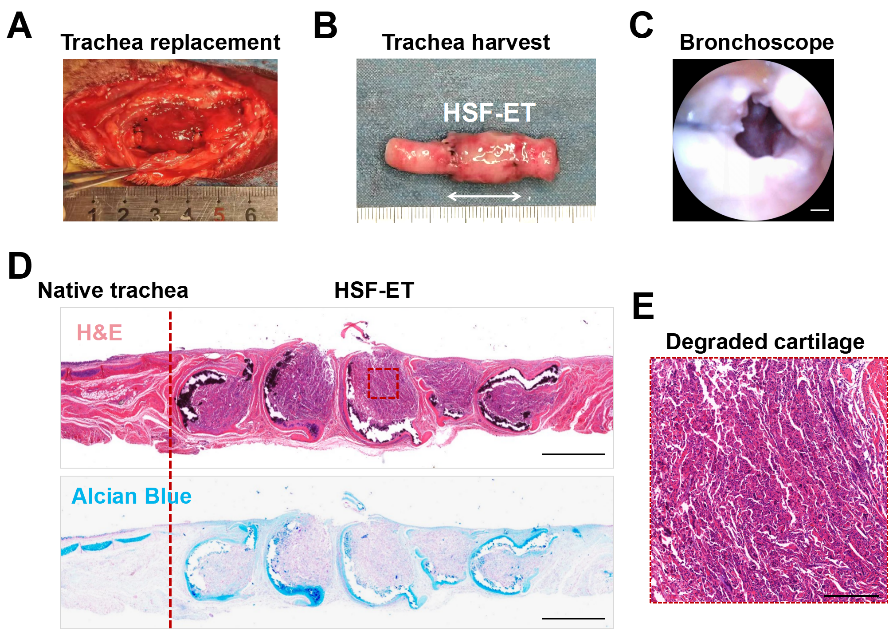


**Figure S18. Evaluation of tissue regeneration of the tracheal substitute without quercetin (HSF-ET). A.** End to end anastomosis of HSF-ET and native rabbit trachea. **B.** Photographs of the trachea sampled after transplantation. **C.** Bronchoscope images. Scale bar: 1mm. **D.** H&E and Alcian blue staining of the representative longitudinal section of HSF-ET. Scale bar: 1.5mm. **E.** Local magnified view of the cartilage degradation area of HSF-ET (H&E staining). Scale bar: 250μm.

**Table S1. The primer sequences of RT-qPCR.**

| **Primer name** | **Primer sequence (5’-3’)** |
| --- | --- |
| COL2 | TGGCAATCCCGGAACAGAC |
|  | CTTGGGACCTTGTTCTCCTTTGA |
| ACAN | TCAACAACAACGCTCAGGACTAC |
|  | TCAAATTGTAGGGGGTGTCCAT |
| COL1 | TCAAATTGTAGGGGGTGTCCAT |
|  | TTATGCCTCTGTCGCCCTGTT |
| COLX | AAGAATGGCACGCCTGTAATGT |
|  | CCTGAGAAGGAGGAATGGACGT |
| CD31 | ACCAAGATAGCCTCAAAGTCGG |
|  | CTGGGAGAGCATTTCACATACG |
| HIF-1α | GCTCATCAGTTGCCACTTCCAC |
|  | CCAAATCACCAGCATCCAGAAG |
| VEGF-A | GGAGGGCAGAATCATCACGA |
|  | GCTCATCTCTCCTATGTGCTGG |

**Movie S1.**

Evaluation of the strength of cartilage rings after being cultured for 4 weeks in vitro.

**Movie S2.**

Mechanical assessment of the cartilage patch after continuous culture in vivo for 4 weeks.

**Movie S3.**

Harvest and tensile test of Hebe-ET after orthotopically transplantation.
